# Patient-Centered Measures of Goal Concordance in Geriatrics and Palliative Care: A Scoping Review

**DOI:** 10.1001/jamanetworkopen.2025.30370

**Published:** 2025-09-04

**Authors:** Isaac S. Chua, Annelise Berler, Kacy Ninteau, Paul A. Bain, Erik K. Fromme, Maria Edelen, Andrea L. Pusic, Christine S. Ritchie, David W. Bates

**Affiliations:** 1 Division of General Internal Medicine and Primary Care, Department of Medicine, Brigham and Women’s Hospital, Boston, Massachusetts; 2 Department of Supportive Oncology, Dana-Farber Cancer Institute, Boston, Massachusetts; 3 Harvard Medical School, Boston, Massachusetts; 4 New England Institute of Clinical Research, Stamford, Connecticut; 5 University of Massachusetts Chan Medical School, Worcester; 6 Countway Library, Harvard Medical School, Boston, Massachusetts; 7 Division of Plastic and Reconstructive Surgery, Department of Surgery, Brigham and Women’s Hospital, Boston, Massachusetts; 8 Mongan Institute Center for Aging and Serious Illness, Division of Palliative Care and Geriatric Medicine, Department of Medicine, Massachusetts General Hospital, Boston; 9 Department of Healthcare Policy and Management, Harvard T.H. Chan School of Public Health, Boston, Massachusetts

## Abstract

**Importance:**

Goal concordance underpins high-quality care for patients with serious illness and older adults with multimorbidity and geriatric syndromes; however, a criterion standard for measurement is lacking.

**Objective:**

To identify and describe patient- and caregiver-reported measures of goal concordance for patients with serious illness, geriatric syndromes, and multimorbidity to create a conceptual model of goal-concordance measurement.

**Evidence Review:**

This scoping review included an electronic search across MEDLINE, Embase, Web of Science Core Collection, the CINAHL, and PsycINFO through September 2024. Search terms included *goals, priorities, preferences, value, expectations, concordance, discordance, match, attain, achieve, align, congruence, dissonance, respect,* and terms related to multimorbidity, geriatric syndromes, and serious illness. The review included randomized clinical trials and quasiexperimental, observational, cohort, cross-sectional, mixed methods, quality-improvement, and qualitative studies, plus secondary analyses that either included a patient- and/or caregiver-reported measure of goal concordance as an outcome or as part of a measure development or validation study. Two reviewers extracted data and resolved disagreements via discussion. Study and measure characteristics were summarized and cross-tabulated to identify measurement framework patterns and to propose a conceptual model of measurement.

**Findings:**

Of 4801 articles screened, 664 full-text articles were assessed for eligibility. A total of 63 studies met inclusion criteria, yielding 67 measures; 44 measures were unique. Most studies were secondary data analyses (17 studies [27.0%]) and included patients with serious illness (44 studies [69.8%]). Measures used 7 of 10 available goal concordance frameworks, with most adopting framework 7 (40 measures [59.7%]), which matched patient preference for an outcome with degree of desired outcome achievement. Four framework components were identified and used to create a conceptual model for goal-concordance measurement: (1) patient preference for an outcome or treatment, (2) treatment undergone or treatment intention, (3) degree of treatment alignment with preference, and (4) degree of desired outcome achievement.

**Conclusions and Relevance:**

This scoping review of patient- and caregiver-reported measures of goal concordance for patients with serious illness, geriatric syndromes, and multimorbidity describes existing measurement frameworks and proposes a conceptual model of measurement that can be used to guide future goal-concordance measure development in geriatrics and palliative care.

## Key Points

**Question:**

What are the characteristics of goal-concordance measurement frameworks currently used in patient- and/or caregiver-reported measures for serious illness, multimorbidity, and geriatric syndromes?

**Findings:**

This scoping review of 63 studies identified 67 measures, of which 44 were unique. Despite measure heterogeneity and preferential adoption of certain frameworks, 4 components of goal-concordance measurement were identified to create an overarching conceptual model for measuring goal concordance.

**Meaning:**

The findings of this study can be used to guide future goal-concordance measure development in geriatrics and palliative care.

## Teaser

This scoping review identifies and describes patient- and caregiver-reported measures of goal concordance for patients with serious illness, geriatric syndromes, and multimorbidity.

## Introduction

Framing care delivery around patients’ goals helps patients prioritize health states that are personally meaningful and simplifies medical decision-making by focusing on outcomes that span conditions while aligning outcomes toward common goals.^1^ This form of patient-centered care has been described as *goal-concordant*^2,3,4^ or *patient priorities–aligned care*.^5,6,7,8^ Goal-concordant, or patient priorities–aligned, care is considered the cornerstone of high-quality care delivery for patients with serious illness and older adults with multimorbidity, including geriatric syndromes.^2,3,4,9,10,11,12,13,14^

A criterion standard for measuring goal-concordant care, or patient priorities-aligned care, remains elusive.^2,3,4^ Additionally, to our knowledge, no structured reviews on this topic exist for older adults with multimorbidity, and reviews in the palliative care literature have been unstructured.^3,4^ One review suggested that measurement of goal-concordant care falls into 4 main methods: (1) patient- or caregiver-reported, (2) postmortem caregiver reporting, (3) concordance in longitudinal data, and (4) population-level indicators.^4^ Although each method has its own strengths and limitations, patient- or caregiver-reported measures hold promise, since such measures may play a larger role in payment and health system reform in the future.^15,16,17^

Therefore, we conducted a scoping review to identify and describe characteristics of patient- and caregiver-reported measures of goal-concordant and patient priorities–aligned care, which we will collectively refer to as *goal concordance* hereafter, for patients with serious illness and older adults with geriatric syndromes and multimorbidity. Specifically, our aims were to (1) describe study and measurement characteristics (including measurement frameworks used to evaluate goal concordance), (2) compare differences in measure characteristics across subpopulations, and (3) create a conceptual model of goal-concordance measurement for patient- and caregiver-reported measures.

## Methods

### Search Strategy and Selection Criteria

We identified studies by searching the electronic databases MEDLINE (Ovid), Embase (Elsevier), Web of Science Core Collection (Clarivate), the Cumulative Index of Nursing and Allied Health Literature (CINAHL, EBSCO), and PsycINFO (EBSCO). The original search was carried out on May 13, 2021, and updated 3 times, with the final search occurring on September 13, 2024. No date limit was applied. This scoping review was reported in accordance with the Preferred Reporting Items for Systematic Reviews and Meta-Analyses extension for Scoping Reviews (PRISMA-ScR).^18^

In the absence of a consensus-based definition,^2,3,4^ we used the National Academy of Medicine’s directive as a starting point for defining goal concordance: “clinicians should work together with patients and their families to ensure that care provided matches closely with each individual’s goals.”^19^ Next, after a brief literature search, we created a list of terms similar to goal concordance to capture measures evaluating equivalent constructs that may have been described differently in other disciplines (eg, *value concordance* in decision science, *goal attainment *in rehabilitation medicine).^20,21,22^ We included the following Medical Subject Headings terms and keywords: *goals, priorities, preferences, value, expectations, concordance, discordance, match, attain, achieve, align, congruence, dissonance*, and* respect *and combined them in various permutations. We also included the following additional terms and keywords to narrow our search related to populations of interest: *advance care planning*, *frail*, *terminally ill*, *palliative care*, *catastrophic illness*, *terminal care*, *hospice*, *multimorbidity*, *multiple chronic conditions*, *aged*, and *geriatrics*, among others. Our full search strategy and rationale are presented in eMethods 1 in Supplement 1.

We included studies that developed, validated, or used patient- and/or caregiver-reported measures that evaluated goal concordance as an outcome. For this review, we were only interested in goal concordance related to patient goals. For example, if a caregiver completed a measure, the measure assessed goal concordance relative to the patient’s goals and not the caregiver’s goals.

Our review concentrated on identifying studies that focused on patients with serious illness and/or multimorbidity and older adults with geriatric syndromes, ie, populations in which goal concordance is essential to high-quality care delivery.^2,3,4,9,10,11,12,13,14^ Because we were interested in identifying and analyzing broader frameworks of goal-concordance measurement and not disease- or condition-specific frameworks, we adopted formal definitions of serious illness, multimorbidity, and geriatric syndromes that allowed for inclusion of a wide number of subpopulations.

We defined *serious illness* based on Kelley et al,^23^ including cancer (metastatic or hematologic), end-stage kidney disease, advanced liver disease or cirrhosis, diabetes with severe complications, amyotrophic lateral sclerosis, AIDS, hip fracture, dementia, chronic obstructive pulmonary disease or interstitial lung disease, and congestive heart failure.^23^ We included the following geriatric syndromes: frailty, polypharmacy, cognitive impairment, falls, incontinence, malnutrition or weight loss, dizziness, and vision or hearing impairment.^24^ We did not use a specific definition of frailty (eg, phenotypic criteria, frailty index)^25^ because most studies lacked such definitions. Therefore, a patient population was considered frail if the study described them as such. We used the numerical definitions of polypharmacy (ie, ≥5 medications daily)^26^ and multimorbidity (ie, ≥2 chronic conditions).^27^

We excluded articles that were not published in English (or if an English translation was unavailable). We also excluded structured literature reviews or original research not published in accordance with PRISMA guidelines,^18^ including study protocols, dissertations or theses, and case studies or series. We excluded studies with populations younger than 18 years, that measured goal concordance in a physical rehabilitation context or setting, assessed goal concordance between participants (eg, patients and caregivers), or measured goal concordance related to a patient’s medical goals (eg, target blood pressure or hemoglobin A_1c_) or self-efficacy (eg, chronic disease management, behavior change). We also excluded measures that assessed patient satisfaction. Detailed inclusion and exclusion criteria are provided in eTable 1 in Supplement 1.

### Screening and Data Abstraction

We used the Covidence systematic review management program to complete the screening process (Veritas Health Innovation). First, we screened articles for relevance based on the information in the title and abstract, followed by determination for inclusion based on the full-text content. Two reviewers (I.S.C. and K.N.) independently screened articles identified during the original search (May 13, 2021) and first update (April 3, 2022). Two reviewers (I.S.C. and A.B.) independently screened articles identified during the second and third updates (July 31, 2023, and September 13, 2024, respectively). Two reviewers (I.S.C. and A.B.) independently abstracted data from all included studies. Disagreements were resolved via discussion and consensus between the 2 reviewers or by a third independent reviewer, as necessary (D.W.B.).

We abstracted the following study data: citation information, funding source, study population category and subcategories, sample size, measure name, measure description completeness, respondent type, goal elicitation method, presence of goal ranking or prioritization, goal-concordance variables (ie, independent or dependent) and measurement framework, goal-concordance determination method, and measure response format. We created classification rules for conditions, syndromes, and diagnoses that overlapped across populations. For example, many older adults and seriously ill patients often have concurrent multimorbidity. However, we classified a study population as patients with multimorbidity only if the study explicitly stated that patients with multimorbidity was a population of interest. We also double-coded studies as geriatric-related syndromes and serious illness if they included patients with dementia and/or cognitive impairment. In studies of dementia and cognitive impairment, researchers often did not report the stage of dementia or degree of cognitive impairment. Therefore, to minimize classification error, we counted studies of dementia as both a geriatric syndrome and serious illness (since the latter must be true if the former is true), but studies of cognitive impairment that did not mention dementia were classified as a geriatric syndrome only.

Reviewers adopted the following protocol for measure inclusion during the full-text review. First, reviewers assessed measures that were explicitly identified as a goal-concordance measure. Next, if none were named, reviewers assessed measures that evaluated patient and/or caregiver experiences related to shared decision-making, patient-centeredness, chronic disease management, goals of care, and/or end of life care. Because these measures were usually not fully described in the article but a reference was often provided, reviewers would review the citations for the measure description. At minimum, included measures were required to contain sections where respondents could designate patient goals and/or rate goal concordance in a structured format. We included measures that lacked respondent ratings of goal concordance if goal concordance could be calculated based on respondent reporting of patient preference and other study data (eg, actual place of death).

Reviewers also used the following criteria to characterize how thoroughly a measure was described. A measure was characterized as completely described when it included an example of the measure as a table or figure in the article or supplemental material or if the measure’s items, response format, and answer choices were described verbatim in the methods. If a measure description was obtained from its citation but not in the original article, the measure was still characterized as completely described. A measure was characterized as partially described if the measure did not meet the criteria for complete description.

We characterized a measure’s goal-concordance measurement framework based on a prior systematic review of value-concordance measurement by Winn et al,^21^ whose construct of value concordance included 3 components: an independent variable, a dependent variable, and model numbers. Unlike Winn et al,^21^ we substituted the term *framework* for *model* and included measures that evaluated respondent perceptions of goal concordance. A description and examples of measurement frameworks included in this review are provided in Table 1, and a full explanation of framework components is provided in eMethods 2 in Supplement 1.

**Table 1.** Components and Examples of Goal Concordance Frameworks

Independent variable^a^	Example^b^	Dependent Variable^c^	Example^d^	Framework No.^e^
Preference for outcomes/attributes of treatments	Patient directly states wanting to be comfortable at the end of life	Treatment undergone	Patient received hospice	1
Treatment preference directly assessed	Patient directly states wanting hospice at the end of life	Treatment undergone	Patient received hospice	2
Treatment preference calculated	Patient answers questions that determined that their preference is most consistent with hospice at the end of life	Treatment undergone	Patient received hospice	3
Preference for outcomes/attributes of treatments	Patient directly states wanting to be comfortable at the end of life	Treatment intention directly assessed	Priority of the patient’s clinician was to provide less aggressive end-of-life care	4
Treatment preference calculated	Patient answers questions that determined that their preference is most consistent with hospice at the end of life	Treatment intention directly assessed	Priority of the patient’s clinician was to provide less aggressive end-of-life care	5
Preference for outcomes/attributes of treatments	Patient directly states wanting to be comfortable at the end of life	Combination of treatment undergone and treatment intention directly assessed	Patient received hospice, and the priority of the patient’s clinician was to provide less aggressive end-of-life care	6
Preference for outcomes/attributes of treatments	Patient directly states wanting to be comfortable at the end of life	Degree of desired outcome achievement^b^	The degree to which the patient achieved comfort at the end of life	7
Preference for outcomes/attributes of treatments	Patient directly states wanting to be comfortable at the end of life	Degree of treatment alignment with preference^c^	The degree to which patient’s wishes were followed in the treatment they received	8
Degree of desired outcome achievement^b^	The degree to which the patient achieved comfort at the end of life	NA	NA	9
Degree of treatment alignment with preference^c^	The degree to which patient’s wishes were followed in the treatment they received	NA	NA	10

Abbrevation: NA, not applicable.

^a^ Independent variables include measure components that ask the respondent to input data regarding the patient’s preferences for health outcomes and/or medical treatments. If a measure does not include such a component and only asks the respondent to evaluate the degree of concordance, then the component evaluating concordance is assigned as the independent variable.

^b^ These are examples of independent variables based on a hypothetical measure that evaluates goal concordance for end-of-life care.

^c^ Dependent variables include measure components that ask the respondent to input data regarding the intention of the treatment the patient will or has received and/or the actual treatment the patient has already received. Dependent variables can also include measure components that ask the respondent to input data on the degree to which treatment aligned with patient preference or to which a desired outcome was achieved. Some studies determined the dependent variable using other collected study data external to the measure.

^d^ These are examples of dependent variables based on a hypothetical measure that evaluates goal concordance for end-of-life care.

^e^ Framework numbers categorize the different combinations of dependent and independent variables.

### Data Synthesis

We summarized study, patient, and measure characteristics of the included articles. To understand patterns of goal-concordance framework adoption across patient populations, we performed a cross-tabulation between measure characteristics and patient groups. For the cross-tabulation, we excluded duplicate measures included across multiple studies. Finally, we created a conceptual model of goal-concordance measurement by mapping the established relationships between independent and dependent variables used in the included measures and proposed potential relationships between framework components based on iterative group consensus.

### Statistical Analysis

Descriptive statistics (frequencies and proportions) and heat maps were generated using Microsoft Excel version 16.97.2 (Microsoft Corporation). Data were analyzed from December 2024 to March 2025.

## Results

### Study Characteristics

A total of 4801 unique articles were identified using the defined search strategy (eMethods 1 in Supplement 1) and screened. Of 664 articles selected for a full-text review, we excluded 601 articles. The most common reasons for exclusion included the study lacked any measure of goal concordance (262 studies), patient population did not meet inclusion criteria (174 studies), and the study lacked a patient- or caregiver-reported measure of goal concordance (57 studies) (eFigure 1 in Supplement 1). A total of 63 articles with 20 630 participants were included in the final review,^28,29,30,31,32,33,34,35,36,37,38,39,40,41,42,43,44,45,46,47,48,49,50,51,52,53,54,55,56,57,58,59,60,61,62,63,64,65,66,67,68,69,70,71,72,73,74,75,76,77,78,79,80,81,82,83,84,85,86,87,88,89,90^ yielding 67 measures, since 4 studies^42,43,53,88^ used 2 measures. There were 44 unique measures because multiple studies used the same measure.

Most studies were published between 2018 and 2024 (36 studies^30,31,36,37,38,39,40,42,43,44,45,50,51,52,55,58,59,60,61,62,63,64,65,67,68,70,75,76,77,80,82,83,84,85,86,90^ [57.1%]) and were conducted in the United States (32 studies^30,31,32,33,34,35,39,40,42,44,45,47,48,49,50,51,53,56,57,64,65,67,68,70,75,76,77,81,85,86,87,88^ [50.8%]) (Table 2). Most studies were secondary data analyses of randomized clinical trials, observational cohort studies, or quasiexperimental studies (17 studies^36,42,54,58,60,61,62,64,65,69,70,71,74,76,80,84,87^ [27.0%]). Most patients had serious illness only (24 studies^28,31,41,44,45,46,51,52,53,59,60,61,62,63,66,68,75,76,77,80,85,86,87,88^ [38.1%]) or had serious illness plus geriatric syndromes or multimorbidity (20 studies^30,36,38,39,40,42,50,54,64,65,67,70,71,72,73,74,82,89,90^ [31.7%]). The most common serious illness diagnosis was cancer (21 studies^28,30,31,40,46,53,58,59,60,61,62,63,64,65,68,76,80,85,87,88,89^ [33.3%]), and the most common geriatric syndromes were cognitive impairment (16 studies^30,32,36,38,39,42,50,54,67,71,72,73,74,82,85,89^ [25.4%]) and incontinence (14 studies^33,34,35,37,47,48,49,55,56,57,69,78,79,83^ [22.2%]). Data abstraction of study characteristics is presented in eTable 2 in Supplement 1.

**Table 2.** Study and Patient Characteristics

Characteristics	Studies, No. (%) (n = 63)
Publication year	
1996-2003	3 (4.8)
2004-2010	9 (14.3)
2011-2017	15 (23.8)
2018-2024	36 (57.1)
Funding source	
Not reported or unclear	16 (25.4)
Public-sponsored only	15 (23.8)
Nonprofit, institutional, or foundation-sponsored only	13 (20.6)
Industry-sponsored only	4 (6.3)
Nonsponsored	4 (6.3)
Multiple types of funding	11 (17.5)
Region and country	
North America	39 (61.9)
US	32 (50.8)
Canada	7 (11.1)
Europe	15 (23.8)
UK	8 (12.7)
Netherlands	6 (9.5)
France	1 (1.6)
Asia	5 (7.9)
Korea	2 (3.2)
Japan	2 (3.2)
Thailand	1 (1.6)
Multiple countries	4 (6.3)
Sample size, No. of patients	
10-100	16 (25.4)
101-200	14 (22.2)
201-500	21 (33.3)
501-1000	9 (14.3)
>1000	3 (4.8)
Study Design	
Secondary data analysis^a^	17 (27.0)
Quasiexperimental	
Overall	13 (20.6)
With mixed methods	1 (1.6)
With application development	1 (1.6)
Observational cohort	12 (19.0)
Randomized clinical trial	
Overall	9 (14.3)
With mixed methods	2 (3.1)
Mixed methods only	5 (7.9)
Quality improvement	
Overall	3 (4.8)
With mixed methods	1 (1.6)
Cross-sectional	4 (6.3)
Role of measure in study	
Outcome (primary or secondary)	56 (88.9)
Measure generation	5 (7.9)
Both	2 (3.2)
Population category	
Serious illness only	24 (38.1)
Geriatric syndromes only	17 (27.0)
Multimorbidity only	2 (3.2)
Serious illness and geriatric syndromes	15 (23.8)
Serious illness and multimorbidity	5 (7.9)
Serious illness subcategory^b^	
Cancer	21 (33.3)
Dementia	15 (23.8)
Heart	7 (11.1)
Kidney	7 (11.1)
Lung	6 (9.5)
Liver	6 (9.5)
Not specified	6 (9.5)
Diabetes and related complications	4 (6.3)
Cerebrovascular accident	3 (4.8)
Other	2 (3.2)
Geriatric syndrome subcategory^b^	
Cognitive impairment	16 (25.4)
Incontinence	14 (22.2)
Frailty	2 (3.2)
Polypharmacy	1 (1.6)

^a^ The primary study designs included randomized clinical trials, observational cohort, or quasiexperimental studies.

^b^ Total percentage will not add up to 100, since subcategories are not mutually exclusive.

### Measure Characteristics

Among 67 measures, approximately two-thirds of measures (45 measures^28,29,30,36,37,39,40,42,43,44,45,46,51,53,54,55,56,58,59,60,61,62,63,64,65,67,70,71,72,73,75,76,77,78,80,81,82,83,84,85,86,87,88,90^ [67.2%]) were fully described (Table 3). Most measured goal concordance as the primary construct,^29,31,32,33,34,35,36,37,38,39,40,41,42,43,44,45,47,48,49,50,51,52,53,54,55,56,57,58,59,60,61,62,63,64,65,66,67,68,69,70,71,72,73,74,75,76,78,79,80,81,82,84,85,86,87,88,89^ but 7 measures^28,30,43,46,77,83,90^ (10.4%) measured goal concordance as part of a larger construct (eg, decision quality, good death). Most measures (33 measures^29,33,34,35,37,40,41,43,47,48,49,51,55,56,57,58,59,60,61,64,65,69,70,76,78,79,80,81,83,84,86,90^ [49.3%]) were solely patient-reported measures and elicited patient goals via free-text responses (24 measures^32,33,36,37,38,43,47,48,49,54,55,56,57,62,63,66,71,72,73,74,78,79,81,82^ [35.8%]), selecting prespecified goals (19 measures^31,40,41,42,51,52,58,59,60,61,64,65,68,70,75,80,83,86,89^ [28.4%]), or both (8 measures^34,35,50,69,76,84,85,88^ [11.9%]). However, 13 measures^28,29,30,39,43,45,46,53,67,77,87,88,90^ (19.4%) measured goal concordance without any goal elicitation (ie, frameworks 9 and 10).

**Table 3.** Measure Characteristics

Characteristics	Measures, No. (%)^a^ (n = 67)
Degree of measure description in study	
Full description or example	45 (67.2)
Partial description or example	22 (32.8)
Role of goal concordance in measure	
Primary construct	60 (89.6)
Secondary construct	7 (10.4)
Respondent type	
Patient only	33 (49.3)
Patient and caregiver^b^	17 (25.4)
Caregiver only	17 (25.4)
Method of goal elicitation	
Free-text goals	
Overall	24 (35.8)
Without preset categories	21 (31.3)
With preset categories	3 (4.5)
Prespecified goals	19 (28.4)
Not applicable	13 (19.4)
>1 Method	
Overall	10 (14.9)
Free text and prespecified goals	8 (11.9)
Free text and prespecified goals and not applicable	1 (1.5)
Prespecified goals and not applicable	1 (1.5)
Unclear	1 (1.5)
Presence of goal ranking or prioritization	
Yes	22 (32.8)
No or not applicable	45 (67.2)
Goal-concordance framework^c^	
1	9 (13.4)
2	1 (1.5)
3	0
4	0
5	0
6	0
7	40 (59.7)
8	1 (1.5)
9	4 (6.0)
10	9 (13.4)
>1 Framework	
Overall	3 (4.5)
2 and 4	1 (1.5)
2, 7, 9, 10, and Other^d^	1 (1.5)
10 and Other^d^	1 (1.5)
Variables reported by respondents^e^	
Independent and dependent variables	48 (71.6)
Independent variable only	17 (25.4)
Unclear	3 (4.5)
Determining goal concordance	
Respondent report only^f^	37 (55.2)
Calculation only	
Overall	26 (38.8)
Matching	17 (25.4)
Measure-specific formula	7 (10.4)
Compared means^g^	1 (1.5)
Calculating agreement^h^	1 (1.5)
>1 Method	
Overall	2 (3.0)
Matching and self-report	1 (1.5)
Matching and regression	1 (1.5)
Unclear	2 (3.0)
Response format when determining goal concordance	
Rating scale^i^	49 (73.1)
Forced choice	7 (10.4)
Multiple choice	4 (6.0)
Binary	2 (3.0)
>1 Format	3 (4.5)
Binary and visual analogue scale	1 (1.5)
Binary, multiple choice, and Likert scale	1 (1.5)
Forced choice and Likert scale	1 (1.5)
Unclear	2 (3.0)

^a^ Total number of measures equals 67 instead of 63 because 4 studies included 2 measures.

^b^ Patient and caregiver completed separate parts of the same measure, 2 different measures to create a composite measure, or the same measure independently.

^c^ Framework numbers categorize the different combinations of dependent and independent variables. Framework components and examples are provided in Table 1.

^d^ Refers to items that ask questions related to goal concordance that do not adhere to the independent-dependent variable framework.

^e^ Total percentage is greater than 100%, since 1 measure used 2 different frameworks to measure goal concordance.

^f^ Some studies created scores or cutoffs to determine what qualifies as goal concordance or summarized sample responses using descriptive statistics. We did not consider such reporting of data as calculation.

^g^ Eg, analysis of variance.

^h^ Eg, κ statististic.

^i^ Includes Likert, numeric rating, visual analogue, and goal attainment scales.

Measures used 7 of 10 available goal-concordance frameworks, with the most widely used being framework 7 (40 measures^31,32,33,34,35,36,37,38,41,43,47,48,49,50,53,54,55,56,57,58,59,60,61,62,63,66,69,71,72,73,74,76,78,79,80,81,82,84,85,88^ [59.7%]) (Table 3). Three measures adopted more than 1 framework; 2 of which included frameworks that did not adhere to the independent-dependent variable framework we adopted (which we classified as other).^42,44^ Most measures did not require any calculation (37 measures^28,29,30,32,33,34,37,39,41,42,43,45,46,47,48,49,50,51,53,55,56,57,62,63,66,67,69,76,77,78,79,82,84,87,88,90^ [55.2%]) to determine goal concordance and relied solely on respondent reporting. However, among 26 measures that calculated goal concordance, matching (which compared respondent preferences with observed or respondent perceptions of outcomes or treatment at 2 time points without additional statistical analyses) was most common (17 measures^31,40,52,53,58,59,60,61,64,65,68,70,75,80,85,86,88^ [65.4%]). Measures mostly used rating scales to obtain respondent input (49 measures^28,29,30,32,33,34,35,36,37,38,39,41,43,45,46,47,48,49,50,51,54,56,57,58,59,60,61,62,63,66,67,69,71,72,73,74,76,77,78,79,80,81,82,83,84,86,88,90^ [73.1%]). Data abstraction of measure characteristics is presented in eTable 3 and eTable 4 in Supplement 1.

Of 67 measures included in the review, 43 (64.2%) were named, of which 16^28,30,31,33,34,35,36,37,38,40,41,42,43,46,47,48,50,52,53,54,55,58,59,60,61,64,65,67,68,69,70,71,72,73,74,75,76,77,80,81,84,90^ were unique (eTable 5 in Supplement 1). Among all studies, the most used measure across all groups was Goal Attainment Scaling (11 studies^33,36,38,43,50,54,71,72,73,74,84^ [17.5%]), which adopted framework 7 and mostly assessed goal concordance for patients with dementia or cognitive impairment.

### Goal-Concordance Frameworks Among Unique Measures Across Patient Populations

**Figure 1.**
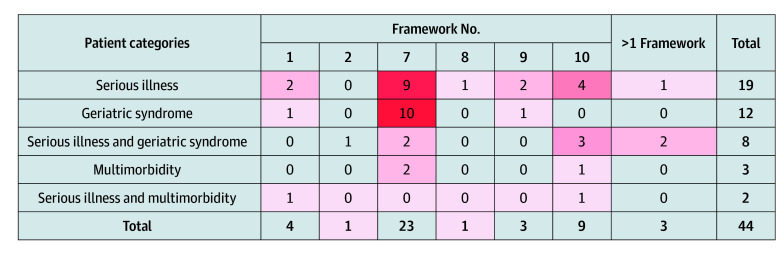
Cross-Tabulation of Goal-Concordance Frameworks Among Unique Measures With Patient Categories Heat map representing the frequency of framework use within a patient category where increased frequency corresponds with darker shades of red. Framework numbers categorize the different combinations of dependent and independent variables.

Among all unique measures, framework 7 was the most common goal-concordance framework incorporated into measures across all populations, except for studies focused on patients with serious illness and multimorbidity (Figure 1).^31,32,33,34,35,36,37,38,41,43,47,48,49,50,53,54,55,56,57,58,59,60,61,62,63,66,69,71,72,73,74,76,78,79,80,81,82,84,85,88^ Among 50 unique measures used across all serious illnesses, framework 7 was used most often for patients with cancer (5 measures [10.0%]) (eFigure 2 in Supplement 1),^28,30,31,40,46,53,58,59,60,61,62,63,64,65,68,76,80,85,87,88,89^ and among 23 unique measures used across all geriatric syndromes, framework 7 was used most often for patients with incontinence (9 measures [39.1%]).^33,34,35,37,47,48,49,55,56,57,69,78,79,83^ Goal-concordance measures for patients with cancer^28,30,31,40,46,53,58,59,60,61,62,63,64,65,68,76,80,85,87,88,89^ and cognitive impairment^30,32,36,38,39,42,50,54,67,71,72,73,74,82,85,89^ incorporated the widest variety of goal-concordance measurement frameworks (eFigure 2 in Supplement 1).

### A Proposed Conceptual Model for Goal-Concordance Measurement

**Figure 2.**
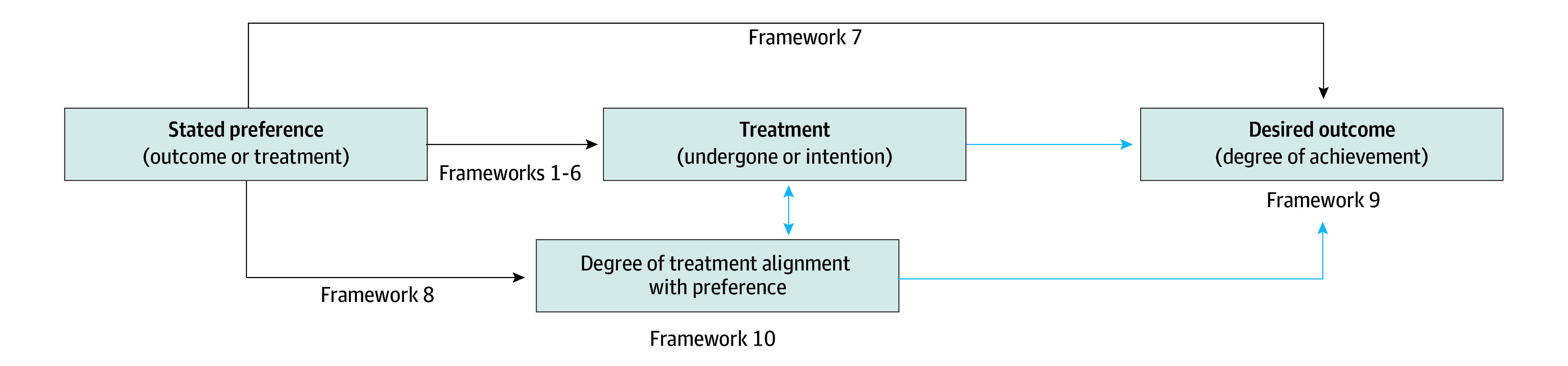
Conceptual Model for Goal-Concordance Measurement Using Patient- and/or Caregiver-Reported Measures The arrows represent measurement frameworks used among measures described and identified in this review. The blue arrows represent hypothetical relationships between framework components that require further validation.

We identified 4 main components of goal-concordance measurement used in patient- and caregiver-reported measures: (1) patient preference for an outcome or treatment, (2) treatment undergone or intention of treatment, (3) degree of treatment alignment with preference, and (4) degree of achieving a desired outcome. Figure 2 depicts a conceptual framework that describes the established relationships between framework components used by measures included in this review and identifies pathways between components that represent future opportunities for measurement and validation.

## Discussion

In this scoping review of patient- and caregiver-reported measures of goal concordance for patients with serious illness, geriatric syndromes, and multimorbidity, we described study and measure characteristics, current frameworks of goal-concordance measurement, and proposed a conceptual model of measurement based on current frameworks. An increasing number of studies have used patient- and/or caregiver-reported measures of goal concordance in recent years. Most studies in this review were conducted in the US and focused on patients with serious illness, especially cancer and dementia. Most measures did not require complex calculations to determine goal concordance and adopted framework 7.

To our knowledge, this is the first scoping review of patient-reported and/or caregiver-reported measures of goal concordance concentrating on patients with serious illness, geriatric syndromes, and/or multimorbidity. Prior systematic reviews of value concordance have included such patient populations but not as the primary population of interest.^21,22^ Compared with similar patient populations in these prior reviews,^91,92,93,94,95,96,97,98^ measures in this scoping review also adopted a wide variety of goal-concordance frameworks. In contrast, measures in this scoping review adopted simpler measurement frameworks that did not calculate treatment preference (ie, frameworks 3 and 5).

Also, in contrast to prior reviews,^21,22^ our scoping review included measures that reported respondent perceptions of goal concordance, which allowed us to describe goal-concordance frameworks not previously described in these reviews (ie, frameworks 7 through 10). These frameworks expand the construct of goal-concordance measurement beyond the preference-treatment relationship used to define value concordance in the decision science literature^21,22^ by introducing 2 components: the degree of achieving a desired outcome (frameworks 7 and 9) and the degree of treatment alignment with preference (frameworks 8 and 10). Our conceptual model integrates these components with the preference-treatment framework and identifies pathways that require further validation and may represent future opportunities for measurement. For example, greater clarity is needed to understand the equivalence between treatment alignment and treatment undergone or intention in this pathway and the relationship between treatment undergone or intention and alignment with desired outcome attainment.

This review includes heterogeneous measures and preferential adoption of goal-concordance measurement frameworks used in patients with serious illness, multimorbidity, and geriatric syndromes. Although the popularity of framework 7 suggests that it may be well-suited for measuring goal concordance in geriatrics and palliative care, framework 7 only represents 1 pathway in our larger conceptual model. Consequently, future research will need to assess the validity and robustness of each measurement pathway within the model. Hopefully, by identifying the most robust pathways in the model, measurement heterogeneity will decrease as future psychometricians will design measures according to the most valid pathways for a population of interest.

### Limitations

This scoping review has several limitations. First, we may have missed measures assessing goal concordance among articles that did not include our search terms as their keywords, especially studies that were published before goal concordance was recognized as a distinct measurement construct. Second, we may have overlooked other measures that may have assessed goal concordance as a secondary construct. Third, we excluded measures of satisfaction, as we felt this was an adjacent construct. One such example is the Canadian Health Care Evaluation Project measure,^99^ which primarily measures end-of-life care experience but includes an item measuring satisfaction with goal concordance. Fourth, we included a variety of search terms to optimize catchment of constructs equivalent or synonymous with goal concordance; however, this may have led to inclusion of measures evaluating a construct that other reviewers may consider goal concordance–adjacent since inclusion was based on the reviewers’ discretion. Fifth, inconsistent measure description across studies by the same authors may have led to overcounting the number of unique measures, especially unnamed measures. Sixth, our classification methods may have overestimated and underestimated the number of measures used in patients with cognitive impairment and patients with multimorbidity, respectively. Seventh, our conceptual model of measuring goal concordance based on existing frameworks of patient- and caregiver-reported measures will require further validation via primary qualitative research, including input from stakeholder groups and expert opinion.

## Conclusions

In this scoping review of patient- and caregiver-reported measures, we discovered heterogeneity and preferential adoption of certain goal-concordance measurement frameworks, some of which may be well-suited for measuring goal concordance in geriatrics and palliative care. In doing so, we identified 4 components of patient- and/or caregiver-reported goal-concordance measurement to create a conceptual model that can be used as the foundation for developing future measures in geriatrics and palliative care.

## Notes

Supplement 1. **eTable 1.** Inclusion and exclusion criteria **eTable 2.** Data abstraction of study characteristics **eTable 3.** Data abstraction of measure characteristics (part 1) **eTable 4.** Data abstraction of measure characteristics (part 2) **eTable 5.** Summary of established goal-concordance measures **eFigure 1.** Preferred reporting items for systematic reviews and meta-analyses (PRISMA) flow **eFigure 2.** Heat map of goal-concordance frameworks among unique measures stratified by serious illness and geriatric syndrome **eMethods 1.** Search strategy and rationale **eMethods 2.** Definitions and provenance of framework components
eReferences.

Supplement 2.
Data Sharing Statement
