# Supplementary material for: Patient-Centered Measures of Goal Concordance in Geriatrics and Palliative Care: A Scoping Review
Source: JAMA Netw Open. 2025 Sep 4;8(9):e2530370. doi: 10.1001/jamanetworkopen.2025.30370 (PMC12411976; doi:10.1001/jamanetworkopen.2025.30370)
Supplement: Supplement 1. — eTable 1. Inclusion and exclusion criteria eTable 2. Data abstraction of study characteristics eTable 3. Data abstraction of measure characteristics (part 1) eTable 4. Data abstraction of measure characteristics (part 2) eTable 5. Summary of established goal-concordance measures eFigure 1. Preferred reporting items for systematic reviews and meta-analyses (PRISMA) flow eFigure 2. Heat map of goal-concordance frameworks among unique measures stratified by serious illness and geriatric syndrome eMethods 1. Search strategy and rationale eMethods 2. Definitions and provenance of framework components eReferences. [file jamanetwopen-e2530370-s001.pdf]

Supplemental Online Content

Chua IS, Berler A, Ninteau K, et al. Patient-centered measures of goal concordance in geriatrics and palliative care: a scoping review. *JAMA Netw Open*. 2025;8(9):e2530370. doi:10.1001/jamanetworkopen.2025.30370

eTable 1. Inclusion and exclusion criteria
eTable 2. Data abstraction of study characteristics
eTable 3. Data abstraction of measure characteristics (part 1)
eTable 4. Data abstraction of measure characteristics (part 2)
eTable 5. Summary of established goal-concordance measures
eFigure 1. Preferred reporting items for systematic reviews and meta-analyses (PRISMA) flow
eFigure 2. Heat map of goal-concordance frameworks among unique measures stratified by serious illness and geriatric syndrome
eMethods 1. Search strategy and rationale
eMethods 2. Definitions and provenance of framework components
eReferences.

This supplemental material has been provided by the authors to give readers additional information about their work.

© 2025 Chua IS et al. *JAMA Network Open*.

eTable 1. Inclusion and exclusion criteria

<table>
<tr><th>Include</th><th>Exclude</th></tr>
<tr><td rowspan="2">Patients ≥18 years old and are:
• Seriously ill
• Have multiple chronic conditions (>1 chronic condition)
• Hospice patients are at the end of life
• Older adults (mean age ≥ 60 years old) with one of the following geriatric syndromes:
  ○ Frailty
  ○ Cognitive impairment
  ○ Falls
  ○ Incontinence
  ○ Low BMI
  ○ Dizziness
  ○ Vision impairment
  ○ Hearing impairment
  ○ Polypharmacy</td><td>Children and young adults (patients < 18 years old)</td></tr>
<tr><td>Adult patients ≥ 18 years old who meet the inclusion criteria but are undergoing rehabilitation.</td></tr>
<tr><td>Inpatient, outpatient, community, and home settings</td><td>Rehabilitation settings</td></tr>
<tr><td rowspan="2">Peer-reviewed articles that describe original research that include ≥1 patient- and/or caregiver-reported measure of goal concordance as an outcome measure or as part of a measure development or validation study.</td><td>Not published in the English language or English translation is unavailable.</td></tr>
<tr><td>Peer-reviewed articles describing a study protocol, case report, case series, dissertation or theses, or structured reviews of the literature</td></tr>
<tr><td>Measurement of goal concordance should assess goals be related to the patient’s personhood. Some examples include, but not limited to:
• Functional ability
• Quality of life
• Length of life
• Connectedness
• Legacy
• Engagement in care
• Shared decision-making
• Symptoms
• Non-specified goals in the context of serious illness, end-of-life, multimorbidity, frailty or geriatric syndromes</td><td>Measurement of goal concordance that focused on medical goals (e.g., blood pressure, hemoglobin A1c) or goals related to self-efficacy (e.g., chronic disease management, behavior change)</td></tr>
<tr><td>Minimum requirements for measure inclusion:</td><td>Measures that measured respondent satisfaction</td></tr>
</table>

© 2025 Chua IS et al. *JAMA Network Open*.

• Respondent(s) designate patient goals, values, priorities, expectations or preferences in a structured manner (i.e., independent variable).*<br>AND/OR<br>• Respondent(s) rate goal concordance in a structured manner.<br><br>*Respondents do not necessarily have to input dependent variables (i.e., treatment undergone, treatment intention assessed, degree of outcome agreement, degree of goal alignment).	

© 2025 Chua IS et al. *JAMA Network Open*.

eTable 2. Data abstraction of study characteristics

First Author (Date)	Article Title	Country	Funding Source	Study population	Sample Size	Serious Illness	Geriatric Syndrome	PMID or DOI
Ahn, E. (2013)	The impact of awareness of terminal illness on quality of death and care decision making: a prospective nationwide survey of bereaved family members of advanced cancer patients	Korea	Public-Sponsored	Serious Illness or End of Life	345	Cancer	N/A	23839783
Bakker, F.C. (2014)	The CareWell in Hospital questionnaire: a measure of frail elderly inpatient experiences with individualized and integrated hospital care	Netherlands	Public-Sponsored	Geriatric syndrome	470	N/A	Frailty	24474635
Bandini, J.I. (2021)	"If we turned our backs, they would ignore our wishes": Bereaved family perceptions of concordance of care at the end of life	United States	Non-profit, Institutional, or Foundation	Geriatric syndrome; Serious Illness or End of Life	601	Cancer; Dementia; Other	Cognitive impairment	33826426
Bernacki, R. (2019)	Effect of the Serious Illness Care Program in Outpatient	United States	Non-profit, Institutional, or Foundation	Serious Illness or End of Life	369	Cancer	N/A	30870563

© 2025 Chua IS et al. *JAMA Network Open*.

First Author (Date)	Article Title	Country	Funding Source	Study population	Sample Size	Serious Illness	Geriatric Syndrome	PMID or DOI
	Oncology: A Cluster Randomized Clinical Trial							
Bogardus, S.T. (2004)	Achieving goals in geriatric assessment: role of caregiver agreement and adherence to recommendations	United States	Not reported	Geriatric syndrome	176	N/A	Cognitive impairment; Frailty	14687322
Bovbjerg, V.E. (2009)	Patient-centered treatment goals for pelvic floor disorders: association with quality-of-life and patient satisfaction	United States	Public-sponsored	Geriatric syndrome	90	N/A	Incontinence	19236871
Brubaker, L. (2011)	Goal attainment scaling in patients with lower urinary tract symptoms: development and pilot testing of the Self-Assessment Goal Achievement (SAGA) questionnaire	United States	Industry-sponsored	Geriatric syndrome	156	N/A	Incontinence	21373818
Brubaker, L. (2013)	Validation study of the Self-Assessment Goal Achievement (SAGA) questionnaire for lower urinary tract symptoms	United States	Industry-sponsored	Geriatric syndrome	104	N/A	Incontinence	23521326

© 2025 Chua IS et al. *JAMA Network Open*.

First Author (Date)	Article Title	Country	Funding Source	Study population	Sample Size	Serious Illness	Geriatric Syndrome	PMID or DOI
Budgett, J (2024)	Personalized goals of people living with dementia and family carers: A content analysis of goals set within an individually tailored psychosocial intervention trial	UK	Non-profit, Institutional, or Foundation	Geriatric syndrome; Serious Illness or End of Life	302	Dementia	Cognitive Impairment	39011459
Cho, M.C. (2018)	Self-assessed goal achievement (SAGA) after Holmium laser enucleation of the prostate (HoLEP): Association with patients' postoperative satisfaction	Korea	Non-sponsored	Geriatric syndrome	170	N/A	Incontinence	30212587
Cooper, C. (2024)	A psychosocial goal-setting and manualised support intervention for independence in dementia (NIDUS-Family) versus goal setting and routine care: a single-masked, phase 3, superiority, randomised controlled trial	UK	Non-profit, Institutional, or Foundation	Geriatric syndrome; Serious Illness or End of Life	247	Dementia	Cognitive impairment	38310894
Cox, C.E. (2018)	Palliative Care Planner: A Pilot	United States	Non-profit, Institutional,	Geriatric syndrome;	171	Stroke / TIA; Liver	Cognitive impairment	29121480

© 2025 Chua IS et al. *JAMA Network Open*.

First Author (Date)	Article Title	Country	Funding Source	Study population	Sample Size	Serious Illness	Geriatric Syndrome	PMID or DOI
	Study to Evaluate Acceptability and Usability of an Electronic Health Records System-integrated, Needs-targeted App Platform		or Foundation	Serious Illness or End of Life		Disease; Heart Disease; Lung Disease; Renal Failure; Dementia		
Curtis, J.R. (2018)	Effect of a Patient and Clinician Communication-Priming Intervention on Patient-Reported Goals-of-Care Discussions Between Patients With Serious Illness and Clinicians: A Randomized Clinical Trial	United States	Non-profit, Institutional, or Foundation	Serious Illness or End of Life; Multimorbidity	618	Liver Disease; Cancer; Heart Disease; Lung Disease; Diabetes + PVD, CAD, or Kidney Disease; Renal Failure	N/A	29802770
de Vries, A.M. (2017)	Goal disturbance changes pre/post-renal transplantation are related to changes in distress	Netherlands	Non-profit, Institutional, or Foundation	Serious Illness or End of Life	220	Renal Failure	N/A	28544010
Ernecoff, N.C. (2018)	Concordance between Goals of Care and Treatment Decisions for Persons with Dementia	United States	Public-sponsored	Geriatric syndrome; Serious Illness or End of Life	302	Dementia	Cognitive impairment	29957095
Ford, J.A. (2019)	Can goal-setting for patients with	UK	Public-sponsored	Multimorbidity	41	N/A	N/A	31164362

© 2025 Chua IS et al. *JAMA Network Open*.

First Author (Date)	Article Title	Country	Funding Source	Study population	Sample Size	Serious Illness	Geriatric Syndrome	PMID or DOI
	multimorbidity improve outcomes in primary care? Cluster randomised feasibility trial							
Glass, D.P. (2021)	Concordance of End-of-Life Care With End-of-Life Wishes in an Integrated Health Care System	United States	Non-profit, Institutional, or Foundation	Serious Illness or End of Life	715	Not specified	N/A	33822069
Haines, L. (2019)	Factors That Impact Family Perception of Goal-Concordant Care at the End of Life	United States	Not reported	Serious Illness or End of Life	1175	Not specified	N/A	30758243
Hirooka, K. (2017)	Quality of death, rumination, and posttraumatic growth among bereaved family members of cancer patients in home palliative care	Japan	Public-sponsored	Serious Illness or End of Life	805	Cancer	N/A	28432854
Hullfish, K.L. (2002)	Patient-centered goals for pelvic floor dysfunction surgery: what is success, and is it achieved?	United States	Not reported	Geriatric syndrome	33	N/A	Incontinence	12114893
Hullfish, K.L. (2004)	Patient-centered goals for pelvic floor dysfunction surgery: long-term follow-up	United States	Not reported	Geriatric syndrome	50	N/A	Incontinence	15295366

© 2025 Chua IS et al. *JAMA Network Open*.

First Author (Date)	Article Title	Country	Funding Source	Study population	Sample Size	Serious Illness	Geriatric Syndrome	PMID or DOI
Hullfish, K.L. (2007)	Colpocleisis for pelvic organ prolapse: patient goals, quality of life, and satisfaction	United States	Public-sponsored	Geriatric syndrome	40	N/A	Incontinence	17666609
Jennings, L.A. (2018)	Personalized Goal Attainment in Dementia Care: Measuring What Persons with Dementia and Their Caregivers Want	United States	Non-profit, Institutional, or Foundation	Geriatric syndrome; Serious Illness or End of Life	133	Dementia	Cognitive impairment	30298901
Kazi, B.S. (2023)	Prevalence and Correlates of Preference-Concordant Care Among Hospitalized People Receiving Maintenance Dialysis	United States	Public-sponsored and Non-profit, institutional, or foundation-sponsored	Serious Illness or End of Life	213	Renal Failure	N/A	37143194
Kentish-Barnes, N. (2024)	A randomised controlled trial of a nurse facilitator to promote communication for family members of critically ill patients	France	Public-sponsored	Serious Illness or End of Life	385	Not Specified	N/A	38573403
Kumar, P. (2017)	Family Perspectives on Hospice Care Experiences of Patients with Cancer	United States	Public-sponsored	Serious Illness or End of Life	2307	Cancer	N/A	27992271

© 2025 Chua IS et al. *JAMA Network Open*.

First Author (Date)	Article Title	Country	Funding Source	Study population	Sample Size	Serious Illness	Geriatric Syndrome	PMID or DOI
Leroi, I. (2014)	Memantine improves goal attainment and reduces caregiver burden in Parkinson's disease with dementia	UK	Public-sponsored	Geriatric syndrome; Serious Illness or End of Life	25	Dementia	Cognitive impairment	24510471
Limbutara, W. (2023)	Patient-reported goal achievements after pelvic floor muscle training versus pessary in women with pelvic organ prolapse. A randomised controlled trial	Thailand	Non-profit, Institutional, or Foundation	Geriatric syndrome	40	N/A	Incontinence	36803636
Mahajan, S.T. (2006)	Patient-centered surgical outcomes: the impact of goal achievement and urge incontinence on patient satisfaction one year after surgery	United States	Not reported	Geriatric syndrome	70	N/A	Incontinence	16522404
Mamik, M.M. (2013)	Goal attainment after treatment in patients with symptomatic pelvic organ prolapse	United States	Not reported	Geriatric syndrome	65	N/A	Incontinence	23770473
Mercadante, S. (2019)	Personalized Goal for Dyspnea and Clinical Response in Advanced Cancer Patients	> 1 country	Non-sponsored	Serious Illness or End of Life	279	Cancer	N/A	30336213

© 2025 Chua IS et al. *JAMA Network Open*.

First Author (Date)	Article Title	Country	Funding Source	Study population	Sample Size	Serious Illness	Geriatric Syndrome	PMID or DOI
Mercadante, S. (2019)	Personalized Symptom Goals and Patient Global Impression on Clinical Changes in Advanced Cancer Patients	> 1 country	Not reported	Serious Illness or End of Life	876	Cancer	N/A	29769382
Mercadante, S. (2020)	Personalized Pain Goals and Responses in Advanced Cancer Patients	> 1 country	Not reported	Serious Illness or End of Life	689	Cancer	N/A	31633792
Mercadante, S. (2021)	The lower the expectations in controlling the symptoms of advanced cancer patients, the better the clinical response	> 1 country	Not reported	Serious Illness or End of Life	876	Cancer	N/A	32926579
Mieras, A. (2020)	Relatives of deceased patients with metastatic lung cancer's views on the achievement of treatment goals and the choice to start treatment: a structured telephone interview study	Netherlands	Other: Non-profit, Institutional, or Foundation and Public-sponsored	Serious Illness or End of Life	118	Cancer	N/A	32560645
Mieras, A. (2021)	Patients with Metastatic Lung Cancer and Oncologists' Views on	Netherlands	Other: Non-profit, Institutional, or Foundation	Serious Illness or End of Life	169	Cancer	N/A	33783264

© 2025 Chua IS et al. *JAMA Network Open*.

First Author (Date)	Article Title	Country	Funding Source	Study population	Sample Size	Serious Illness	Geriatric Syndrome	PMID or DOI
	Achievement of Treatment Goals and Making the Right Treatment Decision: A Prospective Multicenter Study		and Public-sponsored					
Modes, M.E. (2019)	Did a Goals-of-Care Discussion Happen? Differences in the Occurrence of Goals-of-Care Discussions as Reported by Patients, Clinicians, and in the Electronic Health Record	United States	Other: Non-profit, Institutional, or Foundation and Public-sponsored	Serious Illness or End of Life; Multimorbidity	618	Liver Disease; Cancer; Heart Disease; Lung Disease; Diabetes + PVD, CAD, or Kidney Disease; Renal Failure	N/A	30391656
Modes, M.E. (2020)	Patient-Reported Receipt of Goal-Concordant Care Among Seriously Ill Outpatients-Prevalence and Associated Factors	United States	Non-profit, Institutional, or Foundation	Serious Illness or End of Life; Multimorbidity	405	Liver Disease; Cancer; Heart Disease; Lung Disease; Diabetes + PVD, CAD, or Kidney Disease; Renal Failure	N/A	32389606
Needham, P.R. (2004)	Goal setting as a measure of outcome in palliative care	UK	Public-sponsored	Serious Illness or End of Life	104	Not Specified	N/A	15332422

© 2025 Chua IS et al. *JAMA Network Open*.

First Author (Date)	Article Title	Country	Funding Source	Study population	Sample Size	Serious Illness	Geriatric Syndrome	PMID or DOI
Ouchi, K. (2024)	Refinement of an emergency department-based, advance care planning intervention for patients with cognitive impairment and their caregivers	United States	Public-sponsored and Non-profit, institutional, or foundation-sponsored	Geriatric syndrome; Serious Illness or End of Life	26	Dementia	Cognitive impairment	36848220
Prigerson, H.G. (2023)	Advance care planning (ACP) to promote receipt of value-concordant care: Results vary according to patient priorities	United States	Public-sponsored	Serious Illness or End of Life	278	Cancer	N/A	36630471
Rantell, A. (2017)	Personal goals and expectations of OAB patients in the UK	UK	Not reported	Geriatric syndrome	331	N/A	Incontinence	27564470
Rennels, A. (2023)	PREPARE for your care and easy-to-read advance directives increase real-time goal concordant care	United States	Non-profit, institutional, or foundation-sponsored and Public-sponsored	Serious Illness or End of Life; Multimorbidity	798	Stroke / TIA; Liver Disease; Cancer; Heart Disease; Lung Disease; Diabetes + PVD, CAD, or Kidney Disease; HIV/AIDS; Renal Failure; Dementia	N/A	36214201

© 2025 Chua IS et al. *JAMA Network Open*.

First Author (Date)	Article Title	Country	Funding Source	Study population	Sample Size	Serious Illness	Geriatric Syndrome	PMID or DOI
Rockwood, K. (1996)	Use of Goal Attainment Scaling to measure treatment effects in an anti-dementia drug trial	Canada	Industry-sponsored	Geriatric syndrome; Serious Illness or End of Life	15	Dementia	Cognitive impairment	8930946
Rockwood, K. (2002)	Goal setting and attainment in Alzheimer's disease patients treated with donepezil	Canada	Industry-sponsored	Geriatric syndrome; Serious Illness or End of Life	100	Dementia	Cognitive impairment	12397141
Rockwood, K. (2006)	Attainment of treatment goals by people with Alzheimer's disease receiving galantamine: A randomized controlled trial	Canada	Industry-sponsored and Public-sponsored	Geriatric syndrome; Serious Illness or End of Life	128	Dementia	Cognitive impairment	16554498
Rockwood, K. (2007)	Effect of galantamine on verbal repetition in AD: a secondary analysis of the VISTA trial	Canada	Industry-sponsored and Public-sponsored	Geriatric syndrome; Serious Illness or End of Life	130	Dementia	Cognitive impairment	17404193
Rutz Voumard, R. (2021)	Goal-Concordant Care After Severe Acute Brain Injury	United States	Not reported	Serious Illness or End of Life	214	Stroke / TIA	N/A	34603183
Sanders, J.J. (2020)	Measuring Goal-Concordant Care: Results and Reflections From Secondary Analysis of a Trial to Improve	United States	Non-profit, Institutional, or Foundation	Serious Illness or End of Life	203	Cancer	N/A	32599148

© 2025 Chua IS et al. *JAMA Network Open*.

First Author (Date)	Article Title	Country	Funding Source	Study population	Sample Size	Serious Illness	Geriatric Syndrome	PMID or DOI
	Serious Illness Communication							
Saunders, C.H. (2021)	User-Centered Design of the consideRATE Questions, a Measure of People's Experiences When They Are Seriously Ill	United States	Not reported	Serious Illness or End of Life	70	Not specified	N/A	32814165
Srikrishna, S. (2010)	A longitudinal study of patient and surgeon goal achievement 2 years after surgery following pelvic floor dysfunction surgery	UK	Non-sponsored	Geriatric syndrome	112	N/A	Incontinence	20937072
Srikrishna, S. (2015)	Patient and surgeon goal achievement 10 years following surgery for pelvic organ prolapse and urinary incontinence	UK	Non-sponsored	Geriatric syndrome	96	N/A	Incontinence	26089082
Tagami, K. (2020)	The association between health-related quality of life and achievement of personalized symptom goal	Japan	Unclear	Serious Illness or End of Life	140	Cancer	N/A	31970517
Toto, P.E. (2015)	Goal Attainment Scaling (GAS) in geriatric primary	United States	Non-profit, Institutional,	Multimorbidity	25	N/A	N/A	25465505

© 2025 Chua IS et al. *JAMA Network Open*.

First Author (Date)	Article Title	Country	Funding Source	Study population	Sample Size	Serious Illness	Geriatric Syndrome	PMID or DOI
	care: a feasibility study		or Foundation					
Vandermorris, S. (2021)	Understanding the client and family experience of neuropsychological evaluation	Canada	Not reported	Geriatric syndrome; Serious Illness or End of Life	492[a]	Dementia	Cognitive impairment	10.1037pro0000385
vanderWijden, F.C. (2019)	Effectiveness of a web-based treatment decision aid for men with lower urinary tract symptoms due to benign prostatic hyperplasia	Netherlands	Not reported	Geriatric syndrome	217	N/A	Incontinence	30589205
Verdoorn, S. (2019)	The use of goal attainment scaling during clinical medication review in older persons with polypharmacy	Netherlands	Non-profit, institutional, or foundation-sponsored and Industry-sponsored	Geriatric syndrome	315	N/A	Polypharmacy	30425008
Waldrop, D.P. (2020)	"I want to go home": How location at death influences caregiver well-being in bereavement	United States	Not reported	Serious Illness or End of Life	108	Stroke / TIA; Liver Disease; Cancer; Heart Disease; Lung Disease; Renal Failure; Dementia; Other: Neurological diseases,	N/A	32172719

© 2025 Chua IS et al. *JAMA Network Open*.

First Author (Date)	Article Title	Country	Funding Source	Study population	Sample Size	Serious Illness	Geriatric Syndrome	PMID or DOI
						Other/Unknown		
Wallace, B.C. (2021)	Development and piloting of four decision aids for implantable cardioverter-defibrillators in different media formats	United States	Public-sponsored and Non-profit, institutional, or foundation-sponsored	Serious Illness or End of Life	77	Heart Disease	N/A	34528271
Wright, A.A. (2014)	Associations between palliative chemotherapy and adult cancer patients' end of life care and place of death: prospective cohort study	United States	Public-sponsored	Serious Illness or End of Life	386	Cancer	N/A	24594868
Wright, A.A. (2016)	Family Perspectives on Aggressive Cancer Care Near the End of Life	United States	Public-sponsored	Serious Illness or End of Life	1146	Cancer	N/A	26784776
You, J.J. (2014)	What really matters in end-of-life discussions? Perspectives of patients in hospital with serious illness and their families	Canada	Public-sponsored and Non-profit, institutional, or foundation-sponsored	Geriatric syndrome; Serious Illness or End of Life	438	Liver Disease; Cancer; Heart Disease; Lung Disease; Dementia	Cognitive impairment	25367427
Yu, C. (2020)	Impact of MyDiabetesPlan, a Web-Based Patient Decision Aid on Decisional Conflict, Diabetes	Canada	Public-sponsored	Serious Illness or End of Life; Multimorbidity	213	Diabetes + PVD, CAD, or Kidney Disease	N/A	32996893

© 2025 Chua IS et al. *JAMA Network Open*.

First Author (Date)	Article Title	Country	Funding Source	Study population	Sample Size	Serious Illness	Geriatric Syndrome	PMID or DOI
	Distress, Quality of Life, and Chronic Illness Care in Patients With Diabetes: Cluster Randomized Controlled Trial							

[a] Number of subjects was determined based on abstract (i.e., number of individuals who set goals) because it is unclear whether the same or different patients were included in the analysis for goal attainment and patient satisfaction.

© 2025 Chua IS et al. *JAMA Network Open*.

eTable 3. Data abstraction of measures (part 1)

First Author (Date)	Measure Name	Measure Description	Role of Goal-Concordance Construct	Respondent Type	Goal Elicitation Method	Goal Ranking or Prioritization
Ahn, E. (2013)	Good Death Inventory (1 item)	Full	Secondary	Caregiver only	Not applicable	No
Bakker, F.C. (2014)	N/A	Full	Primary	Patient only	Not applicable	No
Bandini, J.I. (2021)	The Toolkit of Instruments to Measure End of Life Care (TIME) (2 items)	Full	Secondary	Caregiver only	Not applicable	No
Bernacki, R. (2019)	Patient Priorities and Family Perceptions Survey	Partial	Primary	Patient and caregiver (interdependent)	Pre-specified only	Yes
Bogardus, S.T. (2004)	N/A	Partial	Primary	Caregiver only	Free-text goals with preset categories	Yes
Bovbjerg, V.E. (2009)	Goal Attainment Scaling	Partial	Primary	Patient only	Free-text goals without preset categories	Yes
Brubaker, L. (2011)	Self-Assessment Goal Achievement (SAGA)	Partial	Primary	Patient only	Both	Yes
Brubaker, L. (2013)	Self-Assessment Goal Achievement (SAGA)	Partial	Primary	Patient only	Both	Yes
Budgett, J (2024)	Goal Attainment Scaling	Full	Primary	Both	Free-text only	No
Cho, M.C. (2018)	Self-Assessed Goal Achievement (SAGA)	Full	Primary	Patient only	Free text goals without preset categories	Yes
Cooper, C. (2024)	Goal Attainment Scaling	Partial	Primary	Both	Free-text only	No
Cox, C.E. (2018)	N/A	Full	Primary	Caregiver only	Not applicable	No
Curtis, J.R. (2018)	SUPPORT question	Full	Primary	Patient only	Pre-specified only	Yes
de Vries, A.M. (2017)	GOALS questionnaire (modified)	Partial	Primary	Patient only	Pre-specified only	Yes

© 2025 Chua IS et al. *JAMA Network Open*.

First Author (Date)	Measure Name	Measure Description	Role of Goal-Concordance Construct	Respondent Type	Goal Elicitation Method	Goal Ranking or Prioritization
Ernecoff, N.C. (2018)	1a) n/a<br><br>1b) n/a	1a) Full<br><br>1b) Full	1a) Primary<br><br>1b) Primary	1a) Caregiver only<br><br>1b) Caregiver only	1a) Pre-specified goals<br><br>1b) Pre-specified goals	1a) Yes<br><br>1b) Yes
	Advance Care Planning Problem Score (2 ad hoc items plus 1 item from the Toolkit After-Death Bereaved Family Member Interview [TIME])	Full	Primary	Caregiver only	Not applicable	No
Ford, J.A. (2019)	Goal Attainment Scaling - Light	Full	Primary	Patient only	Free text goals without preset categories	No
	Patient Assessment of Chronic Illness Care (PACIC) (1 item)	Partial	Secondary	Patient only	Not applicable	No
Glass, D.P. (2021)	N/A (2 versions: Short and Long)	Full	Primary	Caregiver only	Pre-specified goals (Short: Q19, Q30; Long: Q23, Q34)<br><br>Free text goals without preset categories (Short: Q15, Q17; Long: Q19, Q21)<br><br>Not applicable (Short: Q4, Q22, Q24, Q28, Q32; Long Q4, Q6,	No

© 2025 Chua IS et al. *JAMA Network Open*.

First Author (Date)	Measure Name	Measure Description	Role of Goal-Concordance Construct	Respondent Type	Goal Elicitation Method	Goal Ranking or Prioritization
					Q26, Q28, Q32 Q36)	
Haines, L. (2019)	N/A	Full	Primary	Caregiver only	Not applicable	No
Hirooka, K. (2017)	Good Death Inventory (short version) (1 item)	Full	Secondary	Caregiver only	Not applicable	No
Hullfish, K.L. (2002)	Patient Surgical Goals Survey	Partial	Primary	Patient only	Free text goals without preset categories	No
Hullfish, K.L. (2004)	Patient Surgical Goals Survey	Partial	Primary	Patient only	Free text goals without preset categories	No
Hullfish, K.L. (2007)	N/A	Partial	Primary	Patient only	Free text goals without preset categories	No
Jennings, L.A. (2018)	Goal Attainment Scaling	Partial	Primary	Patient and caregiver (interdependent)	Both	Yes
Kazi, B.S. (2023)	N/A	Full	Primary	Patient	Pre-specified only	No
Kentish-Barnes, N. (2024)	N/A	Partial	Primary	Caregiver only	Pre-specified only	Yes
Kumar, P. (2017)	SUPPORT question + N/A	Full	Primary	Caregiver only	Not applicable	No
	N/A	Partial	Primary	Caregiver only	Unclear	No
Leroi, I. (2014)	Goal Attainment Scaling	Full	Primary	Patient and caregiver (interdependent)	Free text goals without preset categories	No
Limbutara, W. (2023)	Self-Assessment Goal Achievement (SAGA)	Full	Primary	Patient only	Free text goals without preset categories	Yes
Mahajan, S.T. (2006)	N/A	Full	Primary	Patient only	Free text goals without preset categories	No

© 2025 Chua IS et al. *JAMA Network Open*.

First Author (Date)	Measure Name	Measure Description	Role of Goal-Concordance Construct	Respondent Type	Goal Elicitation Method	Goal Ranking or Prioritization
Mamik, M.M. (2013)	N/A	Partial	Primary	Patient only	Free text goals without preset categories	Yes
Mercadante, S. (2019)	The Edmonton Symptom Assessment Score (ESAS) with Personalized Dyspnea Intensity Goal	Full	Primary	Patient only	Pre-specified only	No
Mercadante, S. (2019)	Edmonton Symptom Assessment Score (ESAS) + Personalized Symptom Goal (PSG)	Full	Primary	Patient only	Pre-specified only	No
Mercadante, S. (2020)	Edmonton Symptom Assessment Scale + Personalized Pain Goal Response	Full	Primary	Patient only	Pre-specified only	No
Mercadante, S. (2021)	Edmonton Symptom Assessment Score (ESAS) + Patient Symptom Goal (PSG)	Full	Primary	Patient only	Pre-specified only	No
Mieras, A. (2020)	N/A	Full	Primary	Patient and caregiver (interdependent)	Free text goals without preset categories	No
Mieras, A. (2021)	N/A	Full	Primary	Patient and caregiver (independent)	Free text goals without preset categories	No
Modes, M.E. (2019)	SUPPORT Question	Full	Primary	Patient only	Pre-specified only	Yes
Modes, M.E. (2020)	SUPPORT Question	Full	Primary	Patient only	Pre-specified only	No
Needham, P.R. (2004)	N/A	Partial	Primary	Patient and caregiver (independent)	Free text goals without preset categories	No
Ouchi, K. (2024)	Heard and Understood (modified)	Full	Primary	Patient and caregiver (interdependent)	Not applicable	No

© 2025 Chua IS et al. *JAMA Network Open*.

First Author (Date)	Measure Name	Measure Description	Role of Goal-Concordance Construct	Respondent Type	Goal Elicitation Method	Goal Ranking or Prioritization
Prigerson, H.G. (2023)	N/A	Partial	Primary	Patient and caregiver (interdependent)	Pre-specified only	No
Rantell, A. (2017)	Self-Assessment Goal Achievement (SAGA)	Partial	Primary	Patient only	Both	Yes
Rennels, A. (2023)	SUPPORT question	Full	Primary	Patient only	Pre-specified only	Yes
Rockwood, K. (1996)	Goal Attainment Scaling	Full	Primary	Patient and caregiver (interdependent)	Free text goals without preset categories	No
Rockwood, K. (2002)	Goal Attainment Scaling	Full	Primary	Patient and caregiver (interdependent)	Free-text goals with preset categories	Yes
Rockwood, K. (2006)	Goal Attainment Scaling	Full	Primary	Patient and caregiver (interdependent)	Free text goals without preset categories	Yes
Rockwood, K. (2007)	Goal Attainment Scaling	Partial	Primary	Patient and caregiver (interdependent)	Free text goals without preset categories	No
Rutz Voumard, R. (2021)	SUPPORT question	Full	Primary	Caregiver only	Pre-specified only	No
Sanders, J.J. (2020)	Life Priorities Survey	Full	Primary	Patient only	Both	Yes
Saunders, C.H. (2021)	consideRATE	Full	Secondary	Patient and caregiver (independent)	Not applicable	No
Srikrishna, S. (2010)	N/A	Full	Primary	Patient only	Free text goals without preset categories	No
Srikrishna, S. (2015)	N/A	Partial	Primary	Patient only	Free text goals without preset categories	No
Tagami, K. (2020)	Edmonton Symptom Assessment Scale-r (ESAS-r) plus Personalized Symptom Goal (PSG)	Full	Primary	Patient only	Pre-specified only	No

© 2025 Chua IS et al. *JAMA Network Open*.

First Author (Date)	Measure Name	Measure Description	Role of Goal-Concordance Construct	Respondent Type	Goal Elicitation Method	Goal Ranking or Prioritization
Toto, P.E. (2015)	Goal attainment scaling (GAS) and Canadian Occupational Performance Measure (COPM)	Full	Primary	Patient only	Free-text goals with preset categories	Yes
Vandermorris, S. (2021)	N/A	Full	Primary	Patient and caregiver (independent)	Free text goals without preset categories	No
vanderWijden, F.C. (2019)	N/A	Full	Secondary	Patient only	Pre-specified only	Yes
Verdoorn, S. (2019)	Goal Attainment Scaling	Full	Primary	Patient only	Both	No
Waldrop, D.P. (2020)	N/A	Full	Primary	Caregiver only	Both	No
Wallace, B.C. (2021)	N/A	Full	Primary	Patient only	Pre-specified only	Yes
Wright, A.A. (2014)	N/A	Full	Primary	Caregiver only	Not applicable	No
Wright, A.A. (2016)	N/A	Full	Primary	Caregiver only	Not applicable	No
	N/A	Partial	Primary	Caregiver only	Unclear	No
You, J.J. (2014)	N/A	Partial	Primary	Patient and caregiver (independent)	Pre-specified only	No
Yu, C. (2020)	Patient Assessment of Chronic Illness Care (PACIC) (1 item)	Full	Secondary	Patient only	Not applicable	No

© 2025 Chua IS et al. *JAMA Network Open*.

eTable 4. Data abstraction of measures (part 2)

First Author (Date)	Measure Name	Self-Reported Variable	Report Method	Independent	Dependent	Model	Calculation
Ahn, E. (2013)	Good Death Inventory (1 item)	Independent	Likert Scale	Degree of outcome achievement	N/A	9	Self-report only
Bakker, F.C. (2014)	N/A	Independent	Likert Scale	Degree of outcome achievement	N/A	9	Self-report only
Bandini, J.I. (2021)	The Toolkit of Instruments to Measure End of Life Care (TIME) (2 items)	Independent	Likert Scale	Degree of goal alignment	N/A	10	Self-report only
Bernacki, R. (2019)	Patient Priorities and Family Perceptions Survey	Both	Unclear	Preferences for outcomes / attributes	Degree of outcome achievement	7	Matching
Bogardus, S.T. (2004)	N/A	Both	Likert Scale	Preferences for outcomes / attributes	Degree of outcome achievement	7	Self-report only
Bovbjerg, V.E. (2009)	Goal Attainment Scaling	Both	Goal Attainment Scale	Preferences for outcomes / attributes	Degree of outcome achievement	7	Self-report only
Brubaker, L. (2011)	Self-Assessment Goal Achievement (SAGA)	Both	Goal Attainment Scale	Preferences for outcomes / attributes	Degree of outcome achievement	7	Self-report only
Brubaker, L. (2013)	Self-Assessment Goal Achievement (SAGA)	Both	Goal Attainment Scale	Preferences for outcomes / attributes	Degree of outcome achievement	7	Measure-specific formula
Budgett, J (2024)	Goal Attainment Scaling	Both	Goal Attainment Scale	Preferences for outcomes / attributes	Degree of outcome achievement	7	Unclear
Cho, M.C. (2018)	Self-Assessed Goal Achievement (SAGA)	Both	Likert Scale	Preferences for outcomes / attributes	Degree of outcome achievement	7	Self-report only

© 2025 Chua IS et al. *JAMA Network Open*.

First Author (Date)	Measure Name	Self-Reported Variable	Report Method	Independent	Dependent	Model	Calculation
Cooper, C. (2024)	Goal Attainment Scaling	Both	Goal Attainment Scale	Preferences for outcomes / attributes	Degree of outcome achievement	7	Measure-specific formula
Cox, C.E. (2018)	N/A	Independent	Likert Scale	Degree of goal alignment	N/A	10	Self-report only
Curtis, J.R. (2018)	SUPPORT question	Both	Forced Choice	Preferences for outcomes / attributes	Treatment undergone	1	Matching
de Vries, A.M. (2017)	GOALS questionnaire (modified)	Independent	Numerical Rating Scale	Preferences for outcomes / attributes	Degree of outcome achievement	7	Self-report only
Ernecoff, N.C. (2018)	a) n/a<br><br>b) n/a	a) Both<br><br>b) Indpendent	a) Multiple choice<br><br>b) Multiple choice	a) Preferences of outcomes / attributes<br><br>b) Preferences of outcomes / attributes	a) Treatment intention directly assessed<br><br>b) Treatment undergone	a) 4<br><br>b) 2	a) Matching<br><br>b) Regression
	Advance Care Planning Problem Score (2 ad hoc questions plus 1 item from the Toolkit After-Death Bereaved Family Member Interview)	Indpendent	Binary	Other (items #1 and #2) and degree of goal alignment (item #3).	n/a	10 and other	Self-report only
Ford, J.A. (2019)	Goal Attainment Scaling - Light	Both	Goal Attainment Scale	Preferences for outcomes / attributes	Degree of outcome achievement	7	Self-report only
	Patient Assessment of	Independent variable	Likert Scale	Degree of goal alignment	N/A	10	Self-report only

© 2025 Chua IS et al. *JAMA Network Open*.

First Author (Date)	Measure Name	Self-Reported Variable	Report Method	Independent	Dependent	Model	Calculation
	Chronic Illness Care (PACIC) (1 item)						
Glass, D.P. (2021)	N/A (2 versions: Short and Long)	Both	Likert Scale, Binary, Multiple Choice	a) Treatment Preference Directly Assessed (Short: Q15, Q17, Q19; Long: Q19, Q21, Q23)<br><br>b) Preferences for outcomes / attributes (Short: Q30; Long Q34)<br><br>c) Degree of outcome achievement (Short: Q4; Long: Q6)<br><br>d) Degree of goal alignment (Short: Q32; Long: Q36)<br><br>e) Other: Amount of care (Short: Q22, Long: Q26)	a) Treatment undergone (Short: Q14, Q16, Q18; Long: Q18, Q20, Q22)<br><br>b) Degree of outcome achievement (Short: Q20, Long: Q24)	a) 2<br><br>b) 7<br><br>c) 9<br><br>d) 10<br><br>e) Other	a) Matching (Short: Q18/19; Long: Q22/Q23)<br><br>Self-report (Short: Q14/Q15, Q16/Q17; Long: Q18/Q19, Q21/Q22)<br><br>b) Matching<br><br>c) Self-report<br><br>d) Self-report<br><br>e) Self-report

© 2025 Chua IS et al. *JAMA Network Open*.

First Author (Date)	Measure Name	Self-Reported Variable	Report Method	Independent	Dependent	Model	Calculation
				Adequate pain control (Short: Q24, Long: Q28)<br><br>Quality of care in last month (Short: Q28, Long: Q32)<br><br>Satisfied about goal alignment (Long: Q4)			
Haines, L. (2019)	N/A	Independent	Likert Scale	Degree of goal alignment	N/A	10	Self-report only
Hirooka, K. (2017)	Good Death Inventory (short version)	Independent	Likert Scale	Degree of outcome achievement	N/A	9	Self-report only
Hullfish, K.L. (2002)	Patient Surgical Goals Survey	Both	Numerical Rating Scale	Preferences for outcomes / attributes	Degree of outcome achievement	7	Self-report only
Hullfish, K.L. (2004)	Patient Surgical Goals Survey	Both	Goal Attainment Scale	Preferences for outcomes / attributes	Degree of outcome achievement	7	Self-report only
Hullfish, K.L. (2007)	N/A	Both	Likert Scale	Preferences for outcomes / attributes	Degree of outcome achievement	7	Self-report only
Jennings, L.A. (2018)	Goal Attainment Scaling	Both	Goal Attainment Scale	Preferences for outcomes / attributes	Degree of outcome achievement	7	Self-report only
Kazi, B.S. (2023)	N/A	Independent variable	Likert Scale	Degree of goal alignment	N/A	10	Self-report only
Kentish-Barnes, N. (2024)	N/A	Both	Forced Choice	Preferences for outcomes / attributes	Treatment undergone	1	Matching
Kumar, P. (2017)	N/A	Both	Forced Choice and Likert Scale	Preferences for outcomes / attributes	Degree of goal alignment	8	Self-report

© 2025 Chua IS et al. *JAMA Network Open*.

First Author (Date)	Measure Name	Self-Reported Variable	Report Method	Independent	Dependent	Model	Calculation
	N/A	Unclear	Unclear	Preferences for outcomes / attributes	Degree of outcome achievement	7	Matching
Leroi, I. (2014)	Goal Attainment Scaling	Unclear	Goal Attainment Scale	Preferences for outcomes / attributes	Degree of outcome achievement	7	Measure-specific formula
Limbutara, W. (2023)	Self-Assessment Goal Achievement (SAGA)	Both	Binary and Visual Analogue Scale	Preferences for outcomes / attributes	Degree of outcome achievement	7	Self-report only
Mahajan, S.T. (2006)	N/A	Both	Likert Scale	Preferences for outcomes / attributes	Degree of outcome achievement	7	Self-report only
Mamik, M.M. (2013)	N/A	Both	Numerical Rating Scale	Preferences for outcomes / attributes	Degree of outcome achievement	7	Self-report only
Mercadante, S. (2019)	The Edmonton Symptom Assessment Score (ESAS) with Personalized Dyspnea Intensity Goal	Both	Numerical Rating Scale	Preferences for outcomes / attributes	Degree of outcome achievement	7	Matching
Mercadante, S. (2019)	Edmonton Symptom Assessment Score (ESAS) + Personalized Symptom Goal (PSG)	Both	Numerical Rating Scale	Preferences for outcomes / attributes	Degree of outcome achievement	7	Matching
Mercadante, S. (2020)	Edmonton Symptom Assessment Scale plus Personalized Pain Goal Response	Both	Numerical Rating Scale	Preferences for outcomes / attributes	Degree of outcome achievement	7	Matching

© 2025 Chua IS et al. *JAMA Network Open*.

First Author (Date)	Measure Name	Self-Reported Variable	Report Method	Independent	Dependent	Model	Calculation
Mercadante, S. (2021)	Edmonton Symptom Assessment Score (ESAS) + Patient Symptom Goal (PSG)	Both	Numerical Rating Scale	Preferences for outcomes / attributes	Degree of outcome achievement	7	Matching
Mieras, A. (2020)	N/A	Both	Numerical Rating Scale	Preferences for outcomes / attributes	Degree of outcome achievement	7	Self-report only
Modes, M.E. (2019)	N/A	Both	Numerical Rating Scale	Preferences for outcomes / attributes	Degree of outcome achievement	7	Self-report only
Modes, M.E. (2020)	SUPPORT Questionnaire	Both	Forced Choice	Preferences for outcomes / attributes	Treatment undergone	1	Matching
Needham, P.R. (2004)	SUPPORT questionnaire	Both	Forced Choice	Preferences for outcomes / attributes	Treatment undergone	1	Matching
Ouchi, K. (2024)	N/A	Both	Likert Scale	Preferences for outcomes / attributes	Degree of outcome achievement	7	Self-report only
Prigerson, H.G. (2023)	Heard and Understood (modified)	Independent	Likert Scale	Degree of goal alignment	N/A	10	Self-report only
Rantell, A. (2017)	Self-Assessment Goal Achievement (SAGA)	Both	Goal Attainment Scale	Preferences for outcomes / attributes	Degree of outcome achievement	7	Unclear
Rennels, A. (2023)	N/A	Both	Forced Choice	Preferences for outcomes / attributes	Treatment undergone	1	Matching
Modes, M.E. (2019)	SUPPORT questionnaire	Both	Forced Choice	Preferences for outcomes / attributes	Treatment undergone	1	Matching
Rockwood, K. (1996)	Goal Attainment Scaling	Both	Goal Attainment Scale	Preferences for outcomes / attributes	Degree of outcome achievement	7	Measure-specific formula

© 2025 Chua IS et al. *JAMA Network Open*.

First Author (Date)	Measure Name	Self-Reported Variable	Report Method	Independent	Dependent	Model	Calculation
Rockwood, K. (2002)	Goal Attainment Scaling	Both	Goal Attainment Scale	Preferences for outcomes / attributes	Degree of outcome achievement	7	Measure-specific formula
Rockwood, K. (2006)	Goal Attainment Scaling	Both	Goal Attainment Scale	Preferences for outcomes / attributes	Degree of outcome achievement	7	Measure-specific formula
Rockwood, K. (2007)	Goal Attainment Scaling	Both	Goal Attainment Scale	Preferences for outcomes / attributes	Degree of outcome achievement	7	Unclear
Rutz Voumard, R. (2021)	SUPPORT questionnaire	Both	Forced Choice	Preferences for outcomes / attributes	Treatment undergone	1	Matching
Sanders, J.J. (2020)	Life Priorities Survey	Both	Likert Scale	Preferences for outcomes / attributes	Degree of outcome achievement	7	Self-report only
Saunders, C.H. (2021)	consideRATE (1 item)	Independent	Likert Scale	Degree of goal alignment	N/A	10	Self-report only
Srikrishna, S. (2010)	N/A	Both	Visual Analog Scale	Preferences for outcomes / attributes	Degree of outcome achievement	7	Self-report only
Srikrishna, S. (2015)	N/A	Both	Visual Analog Scale	Preferences for outcomes / attributes	Degree of outcome achievement	7	Self-report only
Tagami, K. (2020)	Edmonton Symptom Assessment Scale-r (ESAS-r) plus Personalized Symptom Goal (PSG)	Both	Numerical Rating Scale	Preferences for outcomes / attributes	Degree of outcome achievement	7	Matching
Toto, P.E. (2015)	Goal attainment scaling (GAS) and Canadian Occupational Performance Measure (COPM)	Both	Goal Attainment Scale	Preferences for outcomes / attributes	Degree of outcome achievement	7	Measure-specific formula

© 2025 Chua IS et al. *JAMA Network Open*.

First Author (Date)	Measure Name	Self-Reported Variable	Report Method	Independent	Dependent	Model	Calculation
Vandermorris, S. (2021)	N/A	Both	Likert Scale	Preferences for outcomes / attributes	Degree of outcome achievement	7	Self-report only
Van der Wijden, F.C. (2019)	N/A	Independent	Numerical Rating Scale	Preferences for outcomes/attribute s	Treatment undergone	1	Compared means (e.g., ANOVA)
Verdoorn, S. (2019)	Goal Attainment Scaling	Both	Goal Attainment Scale	Preferences for outcomes / attributes	Degree of outcome achievement	7	Self-report only
Waldrop, D.P. (2020)	N/A	Both	Multiple Choice	Preferences for outcomes / attributes	Degree of outcome achievement	7	Matching
Wallace, B.C. (2021)	N/A	Unclear	Numerical Rating Scale	Preferences for outcomes / attributes	Treatment undergone	1	Matching
Wright, A.A. (2014)	N/A	Independent	Binary (yes/no)	Degree of outcome achievement	N/A	9	Self-report only
Wright, A.A. (2016)	N/A	Independent	Likert Scale	Degree of goal alignment	N/A	10	Self-report only
	N/A	Both	Multiple Choice	Preferences for outcomes / attributes	Degree of outcome achievement	7	Matching
You, J.J. (2014)	N/A	Independent	Multiple Choice	Treatment preference directly assessed	Treatment undergone	2	Calculating agreement (e.g., kappa statistic)
Yu, C. (2020)	Patient Assessment of Chronic Illness Care (PACIC) (1 item)	Independent	Likert Scale	Degree of goal alignment	N/A	10	Self-report only

© 2025 Chua IS et al. *JAMA Network Open*.

eTable 5. Summary of established goal-concordance measures

		Measure Characteristics			Patient Characteristics		
**Measure name**	**Studies, n**	**Framework No.**	**Calculation (n)**	**Respondent (n)**	**Population categories (n)**	**Serious illness subcategories (n)**	**Geriatric syndrome subcategories (n)**
Goal Attainment Scaling (GAS)	11	7	Measure-specific formula (5), Self-report only (4), Unclear (2)	Patient & Caregiver (8) Patient only (3)	Serious illness and Geriatric syndrome (8), Geriatric syndrome only (2), Multimorbidity only (1)	Dementia (8)	Cognitive impairment (8), Incontinence (1), Polypharmacy (1)
SUPPORT question[a]	7	1	Matching (7)	Patient only (4), Caregiver only (2), Patient & Caregiver (1)	Serious illness and multimorbidity (4) Serious illness only (3)	Cancer (5), Heart failure (4), Respiratory failure (4), Liver failure (4), Renal failure (4), Severe diabetes (4) CVA (2), HIV (1) Dementia (1)	N/A
Edmonton Symptom Assessment Scale (ESAS) + personalized goal[b]	5	7	Matching (5)	Patient only (5)	Serious illness only (5)	Cancer (5)	N/A
Self-Assessment Goal Achievement[c]	4	7	Self-report only (3), Measure-specific formula (1)	Patient only (4)	Geriatric syndrome only (4)	N/A	Incontinence (4)
Good Death Inventory (long or short version)[d]	2	9	Self-report only (2)	Caregiver only (2)	Serious illness only (2)	Cancer (2)	N/A
Life Priorities Survey +/- Family Perceptions Survey	2	7	Self-report only (1), Matching (1)	Patient only (1), Patient & Caregiver only (1)	Serious illness only (2)	Cancer (2)	N/A

© 2025 Chua IS et al. *JAMA Network Open*.

Patient Assessment of Chronic Illness Care (PACIC)[d]	2	10	Self-report only (2)	Patient only (2)	Serious illness and Geriatric syndrome (1), Multimorbidity only (1)	Complicated diabetes (1)	N/A
Patient Surgical Goals Survey	2	7	Self-report only (2)	Patient only (2)	Geriatric syndrome only (2)	N/A	Incontinence (2)
The Toolkit of Instruments to Measure End of Life Care (TIME)[e]	1	10	Self-report only (1)	Caregiver only (1)	Serious illness and geriatric syndrome (1)	Cancer (1), Dementia (1), Other/Unknown (1)	Cognitive Impairment (1)
Advance Care Planning Problem Score (TIME[d] + ad hoc items)	1	10 and other	Self-report only (1)	Caregiver only (1)	Serious illness and Geriatric syndrome (1)	Dementia (1)	Cognitive impairment (1)
consideRATE[d]	1	10	Self-report only (1)	Patient & Caregiver (1)	Serious illness only (1)	Not specified (1)	N/A
GAS / Canadian Occupational Performance Measure (COPM)[b]	1	7	Measure-specific formula (1)	Patient only (1)	Multimorbidity only (1)	N/A	N/A
GOALS questionnaire[b]	1	7	Self-report only (1)	Patient only (1)	Serious illness only (1)	Renal failure (1)	N/A
Heard and Understood[b]	1	10	Self-report only (1)	Patient & Caregiver (1)	Serious illness and Geriatric syndrome (1)	Dementia (1)	Cognitive impairment (1)
Self-Assessed Goal Achievement	1	7	Self-report only (1)	Patient only (1)	Geriatric syndrome (1)	N/A	Incontinence (1)
SUPPORT question + ad hoc item	1	8	Self-report only (1)	Patient & Caregiver (1)	Serious illness only (1)	Cancer (1)	N/A

[a] The SUPPORT question is technically not a named measure, but this question is widely used and was commonly referred to as such among the included studies.
[b] These measures were modified from the original version to measure goal concordance.
[c] Measure was derived from Goal Attainment Scaling
[d] 1 item focused on measuring goal concordance as a secondary construct
[e] 2 items focused on measuring goal concordance as a secondary construct

© 2025 Chua IS et al. *JAMA Network Open*.

eFigure 1. Preferred reporting items for systematic reviews and meta-analyses (PRISMA) flow

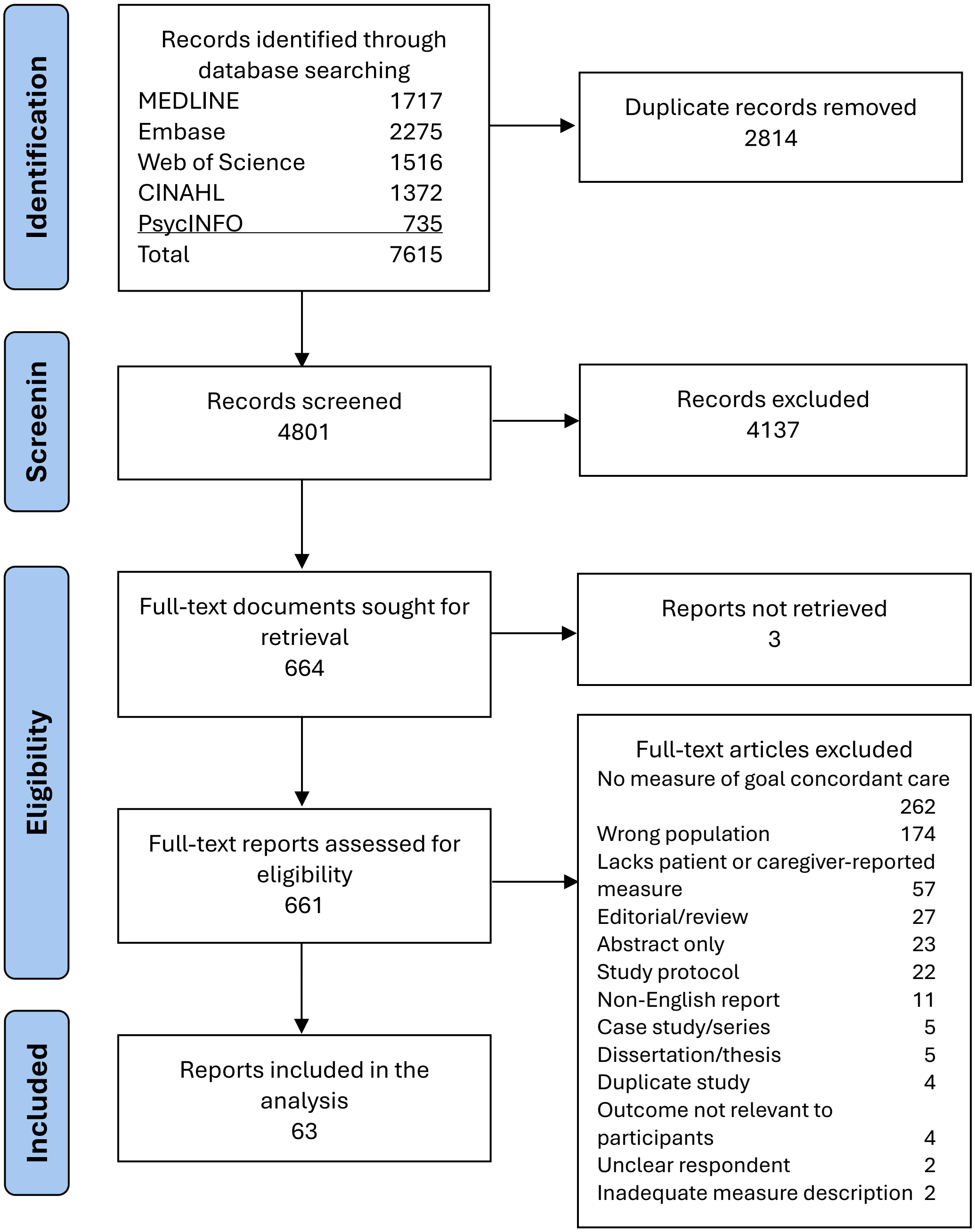

© 2025 Chua IS et al. *JAMA Network Open*.

eFigure 2. Heat map of goal-concordance frameworks among unique measures stratified by serious illness and geriatric syndrome

2a)

	**Framework No.** [a]							
**Serious Illness** [b]	**1**	**2**	**7**	**8**	**9**	**10**	**>1 Framework**	**Total**
Stroke	1	0	1	0	0	1	0	**3**
Liver	1	1	1	0	0	1	0	**4**
Cancer	1	1	5	1	2	2	0	**12**
Heart	2	1	1	0	0	1	0	**5**
Lung	1	1	1	0	0	1	0	**4**
Diabetes	1	0	0	0	0	1	0	**2**
Renal	1	0	2	0	0	2	0	**5**
Dementia	0	1	3	0	0	3	2	**9**
Unknown or Not Specified	1	0	1	0	0	3	1	**6**
**Total**	**9**	**5**	**15**	**1**	**2**	**15**	**3**	**50**

[a] Framework numbers categorize the different combinations of dependent and independent variables.
[b] Some unique measures were used to measure goal concordance in more than one type of serious illness within the same study.

2b)

	**Framework No.** [a]							
**Geriatric Syndrome** [b]	**1**	**2**	**7**	**8**	**9**	**10**	**>1 Framework**	**Total**
Cognitive Impairment	0	1	4	0	0	3	2	10
Incontinence	1	0	9	0	0	0	0	10
Polypharmacy	0	0	1	0	0	0	0	1
Frailty	0	0	1	0	1	0	0	2
**Total**	**1**	**1**	**15**	**0**	**1**	**3**	**2**	**23**

[a] Framework numbers categorize the different combinations of dependent and independent variables.
[b] Some unique measures were used to measure goal concordance in more than one type of geriatric syndrome within the same study.

© 2025 Chua IS et al. *JAMA Network Open*.

eMethods 1. Search Strategy and Rationale

Our rationale for search terminology inclusion was to capture as many synonyms as possible for the term "goal concordance" without losing the essence of what the construct was trying to measure. Because there was no consensus-based or universally accepted definition for goal concordance, we adopted a reductionist approach to the terms that we selected. After a brief literature search, one author (ISC) generated a preliminary list of terms and then received input from two other authors (CSR and DWB) before finalizing the list.

The rubric we adopted was as follows:

1. The first term should be a word that described "what matters to the patient" (e.g., goals, values, preferences, priorities, expectations)
2. The second term should be a word that described "how treatment and care helped accomplish or aligned with what matters to the patient" (e.g., achieved, met, matched, respect, aligned, congruent)

Given the lack of consensus around the definition of goal concordance, we allowed for different combinations of terms to be considered for inclusion. Although some terms may appear to have described different and distinct concepts from each other, the use and combination of terms was highly context specific.

For example, the first terms "goals" and "expectations" seem to describe different concepts but consider a measure that asked the patient to indicate what matters to them about pain control. One measure asks patients to indicate their "*goal* of pain management" and another asks patients to indicate their "*expectation* of pain management." Although slightly different, both measures are asking patients to indicate "what matters to them" regarding pain control.

As another example, the second terms "respect" and "match" may also appear to be describing different concepts but consider a measure that assessed whether "the treatment plan *respected* the patient's wishes about where they wanted to die" versus whether "treatment led the patient to die in a setting that *matched* their preferred place of death." Although the former is measuring treatment alignment with preference and the other is measuring degree of outcome achievement, they are still measuring goal concordance in relation to the patient's preferred place of death.

Ultimately, it was up to the reviewers' discretion as to whether the combined terms equated to "goal concordance," which was determined via discussion between reviewers during the scoping review process.

MEDLINE (Ovid)
Ovid MEDLINE(R) and Epub Ahead of Print, In-Process, In-Data-Review & Other Non-Indexed Citations, Daily and Versions <1946 to September 12, 2024>
September 13, 2024
1717 Records

1. ((goal* or priorit*) adj3 (concord* or discord* or match* or attain* or achiev* or align* or dissonan* or congruen* or respect*)).ab,ti,kf. 50057

2. ((care or value*) adj3 (concord* or discord* or dissonan* or congruen*)).ab,ti,kf. 3540

3. exp Decision Making, Shared/ or (decision* adj3 (share* or making or quality or conflict*)).ab,ti,kf. or decision aid*.ab,ti,kf. 243728

4. patient-centered care/ or (patient* adj3 (centered or centred or experience or focused)).ab,ti,kf. 127876

5. ((goal or priorit*) adj3 (set or setting or level*)).ab,ti,kf. 16975

6. patient preference/ or (patient* adj6 (expectation* or priorit* or preference*)).ab,ti,kf. 63725

© 2025 Chua IS et al. *JAMA Network Open*.

7. exp advance care planning/ or patient care planning/ or (advance* adj3 (plan* or directive)).ab,ti,kf. or living will*.ab,ti,kf. or care plan*.ab,ti,kf. 71768

8. exp aged/ or exp geriatrics/ or (elder* or geriatric* or gerontolog* or frail* or septuagenarian* or octogenarian* or nonagenarian* or centarian* or oldest old or late life).ab,ti,kf. or ((old or older or oldest or aged) adj3 (patient* or adult* or people or individual* or women or men or male* or female* or veteran*)).ab,ti,kf. or ((age or aged or year*) adj3 ("65" or "66" or "67" or "68" or "69" or "70" or "71" or "72" or "73" or "74" or "75" or "76" or "77" or "78" or "79" or "80" or "81" or "82" or "83" or "84" or "85" or "86" or "87" or "88" or "89" or "90" or "91" or "92" or "93" or "94" or "95" or "96" or "97" or "98" or "99" or "100")).ab,ti. 4624755

9. exp terminally ill/ or exp catastrophic illness/ or exp palliative care/ or terminal care/ or exp hospices/ or exp hospice care/ or exp multimorbidity/ or ((terminal* or serious* or catastrophic or advanced or late stage) adj3 (ill* or cancer* or disease*)).ab,ti,kf. or (dying or near death or end stage or "end of life" or palliative or hospic* or terminal care or supportive care or multimorbid* or polymorbid* or multiple chronic condition*).ab,ti,kf. 476572

10. (1 or 2) and (3 or 4 or 5 or 6 or 7) and (8 or 9) 1717

Embase (Elsevier; 1974 -)
September 13, 2024
2275 Records

1. 'goal attainment'/exp OR ((goal* or priorit*) NEAR/3 (concord* or discord* or match* or attain* or achiev* or align* or dissonan* or congruen* or respect*)):ab,ti,kw OR ((care or value*) NEAR/3 (concord* or discord* or dissonan* or congruen*)):ab,ti,kw 56843

2. 'shared decision making'/exp OR (decision* NEAR/3 (share* OR making OR quality OR conflict*)):ab,ti,kw OR 'decision aid*':ab,ti,kw 262069

3 (patient* NEAR/3 (centered OR centred OR experience OR focused)):ab,ti,kw 147961

4. ((goal OR priorit*) NEAR/3 (set OR setting OR level*)):ab,ti,kw 16690

5. 'patient preference'/exp OR (patient* NEAR/6 (expectation* OR priorit* OR preference*)):ab,ti,kw 84165

6. 'advance care planning'/exp OR 'patient care planning'/exp OR 'living will'/exp OR (advance* NEAR/3 (plan* OR directive)):ab,ti,kw OR 'living will*':ab,ti,kw OR 'care plan*':ab,ti,kw 36984

7. aged/exp OR geriatrics/exp OR (elder* OR geriatric* OR gerontolog* OR frail* OR septuagenarian* OR octogenarian* OR nonagenarian* OR centarian* OR 'oldest old' OR 'late life'):ab,ti,kw OR ((old OR older OR oldest OR aged) NEAR/3 (patient* OR adult* OR people OR individual* OR women OR men OR male* OR female* OR veteran*)):ab,ti,kw OR ((age OR aged OR year*) NEAR/3 (65 OR 66 OR 67 OR 68 OR 69 OR 70 OR 71 OR 72 OR 73 OR 74 OR 75 OR 76 OR 77 OR 78 OR 79 OR 80 OR 81 OR 82 OR 83 OR 84 OR 85 OR 86 OR 87 OR 88 OR 89 OR 90 OR 91 OR 92 OR 93 OR 94 OR 95 OR 96 OR 97 OR 98 OR 99 OR 100)):ab,ti 4744542

8. 'terminally ill patient'/exp OR 'terminal disease'/exp OR 'palliative therapy'/de OR 'terminal care'/de OR 'hospice care'/exp OR 'hospice'/exp OR 'multiple chronic conditions'/exp OR ((terminal* OR serious* OR catastrophic OR advanced OR 'late stage') NEAR/3 (ill* OR cancer* OR disease*)):ab,ti,kw OR (dying OR 'near death' OR 'end stage' OR 'end of life' OR palliative OR hospic* OR 'terminal care' OR 'supportive care' OR multimorbid* OR polymorbid* OR 'multiple chronic condition*'):ab,ti,kw 626419

9. #1 AND (#2 OR #3 OR #4 OR #5 OR #6) AND (#7 OR #8) 2275

Web of Science Core Collection (Clarivate Analytics)
Indexes=SCI-EXPANDED, SSCI, A&HCI, CPCI-S, CPCI-SSH, BKCI-S, BKCI-SSH, ESCI, CCR-EXPANDED, IC
Timespan=All years

© 2025 Chua IS et al. *JAMA Network Open*.

September 13, 2024

1516 Records
exact search="on"

1 TS=((("goal*" OR "priorit*") NEAR/3 ("concord*" OR "discord*" OR "match*" OR "attain*" OR "achiev*" OR "align*" OR "dissonan*" OR "congruen*" OR "respect*")) OR (("care" OR "value*") NEAR/3 ("concord*" OR "discord*" OR "dissonan*" OR "congruen*"))) 159409

2 TS=(("decision*" NEAR/3 ("share*" OR "making" OR "quality" OR "conflict*")) OR ("patient*" NEAR/3 ("centered" OR "centred" OR "experience" OR "focused")) OR (("goal" OR "priorit*") NEAR/3 ("set" OR "setting" OR "level*")) OR ("patient*" NEAR/6 ("expectation*" OR "priorit*" OR "preference*")) OR ("advance*" NEAR/3 ("plan*" OR "directive")) OR "living will*" OR "care plan*" OR "decision aid*") 856420

3 TS=("elder*" OR "geriatric*" OR "gerontolog*" OR "frail*" OR "septuagenarian*" OR "octogenarian*" OR "nonagenarian*" OR "centarian*" OR "oldest old" OR "late life" OR (("old" OR "older" OR "oldest" OR "aged") NEAR/3 ("patient*" OR "adult*" OR "people" OR "individual*" OR "women" OR "men" OR "male*" OR "female*" OR "veteran*")) OR (("age" OR "aged" OR "year*") NEAR/3 ("65" OR "66" OR "67" OR "68" OR "69" OR "70" OR "71" OR "72" OR "73" OR "74" OR "75" OR "76" OR "77" OR "78" OR "79" OR "80" OR "81" OR "82" OR "83" OR "84" OR "85" OR "86" OR "87" OR "88" OR "89" OR "90" OR "91" OR "92" OR "93" OR "94" OR "95" OR "96" OR "97" OR "98" OR "99" OR "100"))) 2222668

4 TS=((("terminal*" OR "serious*" OR "catastrophic" OR "advanced" OR "late stage") NEAR/3 ("ill*" OR "cancer*" OR "disease*")) OR "dying" OR "near death" OR "end stage" OR "end of life" OR "palliative" OR "hospic*" OR "terminal care" OR "supportive care" OR "multimorbid*" OR "polymorbid*" OR "multiple chronic condition*") 513408

9 #1 AND #2 AND (#3 OR #4) 1516

CINAHL Complete (EBSCO)
September 13, 2024
1372 Records

1 MH ("Goal Attainment") 2450

2 TI (((goal* OR priorit*) N3 (concord* OR discord* OR match* OR attain* OR achiev* OR align* OR dissonan* OR congruen* OR respect*)) OR ((care OR value*) N3 (concord* OR discord* OR dissonan* OR congruen*))) 2295

3 AB (((goal* OR priorit*) N3 (concord* OR discord* OR match* OR attain* OR achiev* OR align* OR dissonan* OR congruen* OR respect*)) OR ((care OR value*) N3 (concord* OR discord* OR dissonan* OR congruen*))) 18845

4 MH ("Goal-Setting" OR "Decision Making, Shared" OR "Patient Centered Care" OR "Patient Care Plans" OR "Patient Preference" OR "Advance Care Planning") 62185

5 TI ((decision* N3 (share* OR making OR quality OR conflict*)) OR (patient* N3 (centered OR centred OR experience OR focused)) OR ((goal OR priorit*) N3 (set OR setting OR level*)) OR (patient* N6 (expectation* OR priorit* OR preference*)) OR (advance* N3 (plan* OR directive)) OR "living will*" OR "care plan*" OR "decision aid*") 55475

6 AB ((decision* N3 (share* OR making OR quality OR conflict*)) OR (patient* N3 (centered OR centred OR experience OR focused)) OR ((goal OR priorit*) N3 (set OR setting OR level*)) OR (patient* N6 (expectation* OR priorit* OR preference*)) OR (advance* N3 (plan* OR directive)) OR "living will*" OR "care plan*" OR "decision aid*") 166654

© 2025 Chua IS et al. *JAMA Network Open*.

7 MH ("Aged+" OR "Geriatrics") 968169

8 TI (elder* OR geriatric* OR gerontolog* OR frail* OR septuagenarian* OR octogenarian* OR nonagenarian* OR centarian* OR "oldest old" OR "late life" OR ((old OR older OR oldest OR aged) N3 (patient* OR adult* OR people OR individual* OR women OR men OR male* OR female* OR veteran*))) OR AB (elder* OR geriatric* OR gerontolog* OR frail* OR septuagenarian* OR octogenarian* OR nonagenarian* OR centarian* OR "oldest old" OR "late life" OR ((old OR older OR oldest OR aged) N3 (patient* OR adult* OR people OR individual* OR women OR men OR male* OR female* OR veteran*)) OR ((age OR aged OR year*) N3 (65 OR 66 OR 67 OR 68 OR 69 OR 70 OR 71 OR 72 OR 73 OR 74 OR 75 OR 76 OR 77 OR 78 OR 79 OR 80 OR 81 OR 82 OR 83 OR 84 OR 85 OR 86 OR 87 OR 88 OR 89 OR 90 OR 91 OR 92 OR 93 OR 94 OR 95 OR 96 OR 97 OR 98 OR 99 OR 100))) 492767

9 AB (elder* OR geriatric* OR gerontolog* OR frail* OR septuagenarian* OR octogenarian* OR nonagenarian* OR centarian* OR "oldest old" OR "late life" OR ((old OR older OR oldest OR aged) N3 (patient* OR adult* OR people OR individual* OR women OR men OR male* OR female* OR veteran*))) OR AB (elder* OR geriatric* OR gerontolog* OR frail* OR septuagenarian* OR octogenarian* OR nonagenarian* OR centarian* OR "oldest old" OR "late life" OR ((old OR older OR oldest OR aged) N3 (patient* OR adult* OR people OR individual* OR women OR men OR male* OR female* OR veteran*)) OR ((age OR aged OR year*) N3 (65 OR 66 OR 67 OR 68 OR 69 OR 70 OR 71 OR 72 OR 73 OR 74 OR 75 OR 76 OR 77 OR 78 OR 79 OR 80 OR 81 OR 82 OR 83 OR 84 OR 85 OR 86 OR 87 OR 88 OR 89 OR 90 OR 91 OR 92 OR 93 OR 94 OR 95 OR 96 OR 97 OR 98 OR 99 OR 100))) 409391

10 MH ("Terminally Ill Patients" OR "Catastrophic Illness" OR "Palliative Care" OR "Terminal Care" OR "Hospice Care" OR "Hospice Patients" OR "Hospices") 73414

11 TI (((terminal* OR serious* OR catastrophic OR advanced OR "late stage") N3 (ill* OR cancer* OR disease*)) OR (dying OR "near death" OR "end stage" OR "end of life" OR palliative OR hospic* OR "terminal care" OR "supportive care" OR multimorbid* OR polymorbid* OR "multiple chronic condition*") 149550

12 AB (((terminal* OR serious* OR catastrophic OR advanced OR "late stage") N3 (ill* OR cancer* OR disease*)) OR (dying OR "near death" OR "end stage" OR "end of life" OR palliative OR hospic* OR "terminal care" OR "supportive care" OR multimorbid* OR polymorbid* OR "multiple chronic condition*") 166015

13 (S1 OR S2 OR S3) AND (S4 OR S5 OR S6) AND (S7 OR S8 OR S9 OR S10 OR S11 OR S12) 1372

PsycINFO (EBSCO)
September 13, 2024
735 Records

1 TI (((goal* OR priorit*) N3 (concord* OR discord* OR match* OR attain* OR achiev* OR align* OR dissonan* OR congruen* OR respect*)) OR ((care OR value*) N3 (concord* OR discord* OR dissonan* OR congruen*))) 3008

2 AB (((goal* OR priorit*) N3 (concord* OR discord* OR match* OR attain* OR achiev* OR align* OR dissonan* OR congruen* OR respect*)) OR ((care OR value*) N3 (concord* OR discord* OR dissonan* OR congruen*))) 31006

3 DE ("Goal-Setting" OR "Patient Centered Care" OR "Patient Care Plans" OR "Advance Directives" OR "Decision Making") 107353

4 TI ((decision* N3 (share* OR making OR quality OR conflict*)) OR (patient* N3 (centered OR centred OR experience OR focused)) OR ((goal OR priorit*) N3 (set OR setting OR level*)) OR (patient* N6 (expectation* OR priorit* OR preference*)) OR (advance* N3 (plan* OR directive)) OR "living will*" OR "care plan*" OR "decision aid*") 40666

© 2025 Chua IS et al. *JAMA Network Open*.

5 AB ((decision* N3 (share* OR making OR quality OR conflict*)) OR (patient* N3 (centered OR centred OR experience OR focused)) OR ((goal OR priorit*) N3 (set OR setting OR level*)) OR (patient* N6 (expectation* OR priorit* OR preference*)) OR (advance* N3 (plan* OR directive)) OR "living will*" OR "care plan*" OR "decision aid*") 173294

6 AG ("Aged (65 yrs & older)") OR DE ("Geriatrics") 419429

7 TI (elder* OR geriatric* OR gerontolog* OR frail* OR septuagenarian* OR octogenarian* OR nonagenarian* OR centarian* OR "oldest old" OR "late life" OR ((old OR older OR oldest OR aged) N3 (patient* OR adult* OR people OR individual* OR women OR men OR male* OR female* OR veteran*))) OR AB (elder* OR geriatric* OR gerontolog* OR frail* OR septuagenarian* OR octogenarian* OR nonagenarian* OR centarian* OR "oldest old" OR "late life" OR ((old OR older OR oldest OR aged) N3 (patient* OR adult* OR people OR individual* OR women OR men OR male* OR female* OR veteran*)) OR ((age OR aged OR year*) N3 (65 OR 66 OR 67 OR 68 OR 69 OR 70 OR 71 OR 72 OR 73 OR 74 OR 75 OR 76 OR 77 OR 78 OR 79 OR 80 OR 81 OR 82 OR 83 OR 84 OR 85 OR 86 OR 87 OR 88 OR 89 OR 90 OR 91 OR 92 OR 93 OR 94 OR 95 OR 96 OR 97 OR 98 OR 99 OR 100))) 377523

8 AB (elder* OR geriatric* OR gerontolog* OR frail* OR septuagenarian* OR octogenarian* OR nonagenarian* OR centarian* OR "oldest old" OR "late life" OR ((old OR older OR oldest OR aged) N3 (patient* OR adult* OR people OR individual* OR women OR men OR male* OR female* OR veteran*))) OR AB (elder* OR geriatric* OR gerontolog* OR frail* OR septuagenarian* OR octogenarian* OR nonagenarian* OR centarian* OR "oldest old" OR "late life" OR ((old OR older OR oldest OR aged) N3 (patient* OR adult* OR people OR individual* OR women OR men OR male* OR female* OR veteran*)) OR ((age OR aged OR year*) N3 (65 OR 66 OR 67 OR 68 OR 69 OR 70 OR 71 OR 72 OR 73 OR 74 OR 75 OR 76 OR 77 OR 78 OR 79 OR 80 OR 81 OR 82 OR 83 OR 84 OR 85 OR 86 OR 87 OR 88 OR 89 OR 90 OR 91 OR 92 OR 93 OR 94 OR 95 OR 96 OR 97 OR 98 OR 99 OR 100))) 368121

9 DE ("Terminally Ill Patients" OR "Palliative Care" OR "Hospice") 22335

10 TI (((terminal* OR serious* OR catastrophic OR advanced OR "late stage") N3 (ill* OR cancer* OR disease*)) OR (dying OR "near death" OR "end stage" OR "end of life" OR palliative OR hospic* OR "terminal care" OR "supportive care" OR multimorbid* OR polymorbid* OR "multiple chronic condition*") 101076

11 AB (((terminal* OR serious* OR catastrophic OR advanced OR "late stage") N3 (ill* OR cancer* OR disease*)) OR (dying OR "near death" OR "end stage" OR "end of life" OR palliative OR hospic* OR "terminal care" OR "supportive care" OR multimorbid* OR polymorbid* OR "multiple chronic condition*") 112315

12 (S1 OR S2) AND (S3 OR S4 OR S5) AND (S6 OR S7 OR S8 OR S9 OR S10 OR S11) 735

© 2025 Chua IS et al. *JAMA Network Open*.

eMethods 2. Definitions and provenance of framework components

The terms *independent variables, dependent variables,* and *model numbers* in this scoping review were adapted from an updated systematic review (Winn et al.) evaluating how studies defined and reported concordance between patients' preferences and medical treatments, which they defined as *value concordance*.[1] In that systematic review by Winn et al, they defined the terms as follows:

1. *Independent variables* – "patients' preferences concerning health outcomes and/or medical treatments."
2. *Dependent variables* – "treatment intention or treatment undergone."
3. *Value concordance* – relationship between *independent variables* (i.e., preferences for health outcomes and/or treatments) and *dependent variables* (i.e., intended or actual treatments).

For a study to be included in that review, *value concordance* needed to be calculated and reported. The term *model numbers* were used to classify the combinations of *dependent and independent variables*. The table below delineates the models used in Winn et al and describes the different combinations of *dependent* and *independent variables* for each *model number*.[1] We adopted this same method in our review; the only difference is that we used the term "framework number" instead of *model number*.

Independent Variables	Dependent Variables	Model Numbers
Preference for Outcomes / Attributes	Treatment Undergone	1
Treatment Preference Directly Assessed	Treatment Undergone	2
Treatment Preference Calculated	Treatment Undergone	3
Preference for Outcomes / Attributes	Treatment Intention Directly Assessed	4
Treatment Preference Calculated	Treatment Intention Directly Assessed	5
Preference for Outcomes / Attributes	Combination of Treatment Undergone and Treatment Intention Directly Assessed	6

Their method of defining *value concordance* was derived from an earlier systematic review by Sepucha et al.[2] In that systematic review, they used slightly different terms. Instead of *independent variable* and *dependent variable,* they use the terms *preference* and *choice*, respectively. They defined those terms as follows:

1. *Preference* – "patients' preferences concerning health outcomes and/or medical treatments."
2. *Choice* –"patients' intended or actual choices."
3. *Value concordance* – relationship between *preference* and *choice.*

When calculating concordance, they treated *preference* as the independent variable and *choice* as the dependent variable. This method of matching *preference* and *choice* stemmed from an international consensus process, the International Patient Decision Aids Standards (IPDAS) Collaboration, which determined that a high-quality patient decision aid "improves the match between the chosen option and the features that matter most to the informed patient."[3,4]

Upon reviewing the initial full text articles, we realized that solely characterizing a measure based on the relationship between *preference* and *choice* (i.e., treatment) would be inadequate for some measures we included. Therefore, we decided to adopt Winn et al's classification system because the terms *independent variable* and *dependent variable* allowed us to incorporate other components of goal concordance beyond the relationship between preference and choice/treatment.

The subsequent frameworks emerged during the data abstraction phase. Reviewers characterized components based on the type of data the measures were asking respondents to rate or input. During the initial part of the review, the reviewers entered a description of the measure during their independent data abstraction process. When reviewers would meet to discuss disagreements, they would also discuss measure descriptions to identify patterns across measures. Through this inductive process, the following goal-concordance framework components emerged from the data:

1. Preference for outcomes / attributes of treatment(s) – same component from the earlier reviews.

© 2025 Chua IS et al. *JAMA Network Open*.

2. Degree of treatment alignment with preference– how well treatment or care provided was consistent with or aligned with what matters to patients (i.e., process-focused).
3. Degree of achieving a desired outcome – how well patients were able to accomplish or achieve their goals or desired outcome as a result from treatment (i.e., outcome-focused)

When assigning components to variables, we adopted the same approach as Winn et al.[1] and assigned "preferences for outcomes / attributes of treatments" as the *independent variable*. Consequently, "degree of treatment alignment with preference" or "degree of achieving desired outcome" were considered *dependent variables* if patient preferences were also elicited. However, because we also included respondent perceptions of goal concordance, some measures did not specifically ask about patient preferences and only assessed concordance. For these measures, we classified "degree of treatment alignment with preference" and "degree of achieving desired outcome" as *independent variables* because patient preferences were absent.

© 2025 Chua IS et al. *JAMA Network Open*.

eReferences

1. Winn K, Ozanne E, Sepucha K. Measuring patient-centered care: An updated systematic review of how studies define and report concordance between patients' preferences and medical treatments. *Patient Educ Couns.* 2015;98(7):811-821.
2. Sepucha K, Ozanne EM. How to define and measure concordance between patients' preferences and medical treatments: A systematic review of approaches and recommendations for standardization. *Patient Educ Couns.* 2010;78(1):12-23.
3. Elwyn G, O'Connor A, Stacey D, et al. Developing a quality criteria framework for patient decision aids: online international Delphi consensus process. *Bmj.* 2006;333(7565):417.
4. International Patient Decision Aid Standards Collaboration. Original IPDAS checklist (74 items). https://decisionaid.ohri.ca/IPDAS/using.html. Updated March 13, 2024. Accessed July 29, 2025.

© 2025 Chua IS et al. *JAMA Network Open*.
