# Supplementary material for: Patient-Centered Measures of Goal Concordance in Geriatrics and Palliative Care: A Scoping Review
Source: JAMA Netw Open. 2025 Sep 4;8(9):e2530370. doi: 10.1001/jamanetworkopen.2025.30370 (PMC12411976; doi:10.1001/jamanetworkopen.2025.30370)
Supplement: Supplement 2. — Data Sharing Statement [file jamanetwopen-e2530370-s002.pdf]

Data Sharing Statement

Chua. Patient-Centered Measures of Goal Concordance in Geriatrics and Palliative Care.
JAMA Netw Open. Published September 04, 2025. doi:10.1001/jamanetworkopen.2025.30370

Data
Data available: No
